# Eight weeks of focal muscle vibration shows improvements in muscle co-ordination and co-contraction in cerebral palsy children: a randomized control trial

**DOI:** 10.1186/s12984-026-02066-8

**Published:** 2026-07-30

**Authors:** Moeez Ashfaque, Kiran Khushnood, Ganesh R. Naik, Imran K. Niazi, Imran Amjad, Amit N. Pujari

**Affiliations:** 1 Neu(RAL)2: NeuRAL Systems and Rehabilitation and Assistive Technologies Laboratory, School of Physics, Engineering and Computer Science, University of Hertfordshire, Hatfield, AL10 9EU UK; 2 Riphah International University, Islamabad, 46000 Pakistan; 3 College of Medicine and Public Health, Flinders University, Adelaide, Australia; 4 Australia and Design and Creative Technology Vertical, Torrens University, Adelaide, Australia; 5 Centre for Chiropractic Research, New Zealand College of Chiropractic, Auckland, New Zealand; 6 Health and Rehabilitation Research Institute, Auckland University of Technology, Auckland, New Zealand; 7 3SMI, Department of Health Science and Technology, Aalborg University, Aalborg, Denmark; 8 School of Engineering, University of Aberdeen, Aberdeen, AB24 3FX UK

**Keywords:** Cerebral palsy, Focal muscle vibration, Electromyography, Neuromuscular activation, Co-contraction, Fatigue dynamics

## Abstract

**Background:**

Muscle spasticity, impaired coordination and fatigue are common in cerebral palsy (CP) children. This study investigated frequency-specific effects of focal muscle vibration (FMV) on neuromuscular activation, fatigue, and agonist–antagonist coordination. We hypothesized that 8 weeks of FMV would improve lower limb muscle activation, reduce fatigue, and optimize co-contraction compared to control, with differential effects between 80 and 100 Hz stimulation.

**Methods:**

In a double-blind randomized controlled trial, 65 spastic diplegic CP children (GMFCS II–IV) were allocated to Control, 80 Hz, or 100 Hz FMV groups. Primary outcomes were EMG-derived muscle activation (RMS) and fatigue (median frequency; and fatigue index). Secondary outcomes included co-contraction index (CCI) between tibialis anterior (TA) and soleus (Sol). Assessments were conducted at baseline, 4, 8, and 12 weeks. Linear mixed-effects models evaluated Group, Time, and Group × Time effects, with Bonferroni-corrected post-hoc comparisons.

**Results:**

Significant group differences were observed primarily in the TA. In non-dominant leg, the 100 Hz group showed higher RMS compared to Control at 4 weeks (*p* = 0.027) and 8 weeks (*p* = 0.027) and compared to 80 Hz at 4 weeks (*p* = 0.045) and 8 weeks (*p* = 0.008). In dominant leg, 100 Hz also demonstrated higher RMS than Control at 8 weeks (*p* = 0.003). Fatigue analysis revealed significant time effects in soleus muscle (*p* ≤ 0.009), with 100 Hz group demonstrating more sustained MDF values in the dominant limb, suggesting improved fatigue resistance, whereas 80 Hz group showed greater fatigue-related declines. FI analysis further revealed significant Group × Time interactions in non-dominant Sol (*p* = 0.008), with 80 Hz differing from Control (*p* = 0.019) and 100 Hz (*p* = 0.008) at 8 weeks. For co-contraction, a significant group effect was observed in the non-dominant limb (*p* = 0.034), indicating differences between intervention and control groups, although post-hoc comparisons were not significant.

**Conclusion:**

FMV induces frequency-specific neuromuscular adaptations in CP children. 100 Hz stimulation appears more effective for enhancing short-term muscle activation and fatigue resistance, while 80 Hz may promote longer-term coordination changes.

*Trial registration* Trial prospectively registered with United States clinical trial registry (clinicaltrials.gov), trial ID: NCT05751135.

## Highlights

- *Clinical and Impact*: Clinically, the findings advocate personalized FMV protocols and adjunct therapies to prolong benefits. This study advances mechanistic insights into FMV, informing targeted rehabilitation strategies for CP.

## Background

Cerebral palsy (CP) is a non-progressive neurological disorder stemming from early brain insult, and can lead to complications like cognitive deficits, epilepsy, and musculoskeletal deformities [1]. CP is one of the leading causes of childhood motor disability [2], globally affecting 2–3 per 1000 live births approximately [1]. It has a profound socioeconomic burden [3–6], costing up to $2.9 billion annually in direct and $12.2 billion in indirect costs in the US, for example [7]. Spastic CP accounts for around 80% CP cases (35% diplegic, 20% quadriplegic and 25% hemiplegic) [1, 8], and is characterized by hypertonia and stiffness, muscle weakness, impaired coordination, and restricted mobility [1, 8, 9]. Current rehabilitation strategies for CP provide partial relief, require specialized effort, or are invasive. Physical therapy – involving structured exercises, is limited by long-term effectiveness [10, 11], occupational therapy fails to address underlying neurological deficits [12], and pharmacological agents, such as botulinum toxin injections, temporarily reduce spasticity but carry risks of sedation, fatigue, and tolerance development [13–16]. Surgical interventions, including selective dorsal rhizotomy, offer lasting relief for severe spasticity but involve invasive risks and extended recovery [17, 18].

Focal muscle vibration (FMV) has emerged as a promising neuromodulatory intervention for CP. By delivering mechanical vibration stimuli to the targeted muscles, FMV enhances proprioceptive feedback, activates muscle spindles, and may induce cortical reorganization through plasticity [19–23]. Unlike conventional therapies, FMV is passive, non-invasive, and customizable to individual needs via adjustable frequencies and amplitudes [24, 25]. Wearable FMV devices have already shown promising results in improving limb function and reducing spasticity in the stroke population [26, 27].

Focal muscle vibration (FMV) primarily acts through activation of muscle spindle primary endings (Ia afferents), which are highly sensitive to vibration frequencies typically ranging between 60 and 120 Hz [28, 29]. Importantly, the firing behaviour of these afferents is not linear across frequencies; rather, it exhibits frequency-dependent entrainment and saturation characteristics [30, 31]. Experimental neurophysiology studies have shown that Ia afferents can synchronize their discharge to vibration stimuli, with optimal responsiveness often reported around 80–100 Hz, but with subtle shifts in firing regularity, recruitment, and central transmission occurring even within this range [32–34].

Critically, it is not only the mean firing rate but the temporal regularity of this discharge that shapes downstream effects: more regular, phase-locked Ia volleys produce more reliable temporal summation at the motoneuron pool and more coherent corticospinal drive, whereas small upward shifts in frequency near the entrainment ceiling of Ia afferents can push firing toward intermittent or saturated patterns [31, 32, 39, 40]. Because such synchronized, regular afferent input is the principal driver of the long-term potentiation (LTP)-like plasticity thought to underlie durable motor improvements after repeated muscle vibration [65], even modest changes in discharge regularity can alter how efficiently a given frequency consolidates neuromuscular adaptation, rather than determining whether an effect occurs at all.

Small variations in vibration frequency may therefore differentially modulate the temporal pattern of afferent input to the central nervous system. There is growing evidence of FMV’s utility in neurorehabilitation of conditions such as stroke, multiple sclerosis, and spinal cord injury, and the magnitude of therapeutic benefit is consistently tied to the vibration frequency employed [19, 24, 35–37]. For example, [25] found that in spinal injury populations, 80 Hz better modulates reciprocal inhibition than 20, 40, and 120 Hz. Therefore, identifying the optimal stimulation frequency becomes important for effective rehabilitation. Furthermore, evidence from studies on vibration-induced kinesthetic illusions and corticospinal excitability suggests that even small differences in vibration frequency can alter the balance between spinal reflex pathways and supraspinal processing [34, 38, 39]. This may result in frequency-specific effects on motor coordination, co-contraction patterns, and fatigue dynamics. In populations with neurological impairments such as cerebral palsy, where sensorimotor integration is already disrupted, these subtle differences in afferent drive may be amplified, leading to distinct functional outcomes.

Among the most widely reported FMV frequencies for neurorehabilitation are 80 Hz and 100 Hz, both of which fall within the optimal range for primary spindle afferent activation [29, 32–34, 40]. While 100 Hz has been the more extensively studied frequency, predominantly for its acute spasticity reduction and cortical excitability modulation, 80 Hz has been associated with distinct effects on agonist–antagonist coordination through preferential engagement of spinal inhibitory circuits [19, 25, 36, 37]. Despite both frequencies being within 20 Hz of each other, the available evidence suggests they may differ mainly in the magnitude and temporal persistence of their effects rather than engaging qualitatively distinct mechanisms; indeed, perceptual studies report that frequencies above approximately 80 Hz alter mainly the duration of vibratory after-effects rather than their qualitative nature [63]. We therefore did not assume separate mechanisms a priori but instead tested whether 80 Hz and 100 Hz would modulate the size and time course of neuromuscular adaptations differentially in children with spastic diplegic CP, over a sustained 8-week intervention period.

In the CP population, preliminary studies demonstrate FMV’s short-term benefits, including reduced spasticity and improved gait parameters [41–43]. For instance, three days of FMV intervention at 100 Hz has been shown to reduce spasticity up to 40% [20]. However, existing research predominantly focuses on the acute effects or low dosage of FMV; with limited insight into sustained neuromuscular adaptations or (vibration) stimulation frequency-specific mechanisms caused by FMV, limiting FMV’s therapeutic potential.

Crucially, the relationship between vibration parameters (e.g., frequency, duration) and long-term neuroplastic changes remains poorly characterized. While electromyography (EMG) offers a window into neuromuscular dynamics, few studies have employed EMG to quantify how FMV influences muscle activation, fatigue, or agonist–antagonist coordination over extended periods. Present EMG studies focus on using EMG as biofeedback to guide vibration delivery rather than on understanding the effect of vibration on the neuromuscular underlay [43]. Addressing these gaps could refine FMV protocols, optimize outcomes, and unlock its full rehabilitative potential.

Therefore, this study aimed to investigate the efficacy of two FMV frequencies (80 Hz and 100 Hz) administered over 8 weeks in children with spastic diplegic CP. Using surface EMG (sEMG) during dorsiflexion tasks, we evaluated (1) time-dependent changes in muscle activation, (2) fatigue dynamics, and (3) co-contraction between the agonist and antagonist muscles in the lower limb. We hypothesized that FMV stimuli (80 Hz/100 Hz/both) would elicit superior muscle activation patterns and exhibit greater fatigue resilience, compared to the Control (no vibration). By elucidating (stimulation) frequency-specific mechanisms, this work aims to advance FMV as a precision therapy for CP, bridging the gap between empirical application and mechanistic understanding. As per our knowledge, this is the first comprehensive study to systematically investigate the effects of wearable FMV on muscle activation, fatigue and co-contraction, in spastic diplegic CP children. This is also the first study to compare these effects, with two FMV stimulation frequencies (80 and 100Hz) over a longer (12 weeks) period, in CP children. Thus, the study aims to provide more comprehensive insight into the use of wearable FMV as an intervention technology in CP children.

## Methods

### Participants

The inclusion criteria were age between 3 and 15 years, a clinical diagnosis of spastic diplegic CP, Gross Motor Function Classification Scale (GMFCS) levels II-IV, absence of severe cognitive, visual, or hearing impairments, no history of metabolic diseases, and no history of fractures in the past one year. Additionally, individuals who had received botulinum toxin injections within the past year were excluded. The study was designed as a double-blind, randomized, controlled trial with a three-group parallel structure. Participants were randomly assigned to one of three groups: a Control group and two intervention groups, labelled intervention-1a (80 Hz) and intervention-1b (100 Hz). Randomization was computer-generated. Groups were comparable in age, GMFCS level, and MAS scores at baseline. The participants and outcome assessors were kept blinded about the allocated groups. Due to the physical nature of vibration intervention, it was not possible to blind the control group from the intervention groups. The sample size for the main trial was calculated using G*Power, with an effect size of 0.25, a power of 80%, and three groups, resulting in a requirement of 96 participants. However, EMG assessments were only performed in a sub-cohort, due to the limitation in time and resources. Eligible participants for EMG data collection were randomly allocated into groups. Recruitment was open for a maximum of two months, during which we aimed to enrol as many eligible participants as possible. The two-month recruitment period was determined by the availability of researchers and equipment on site.

**Fig. 1:**
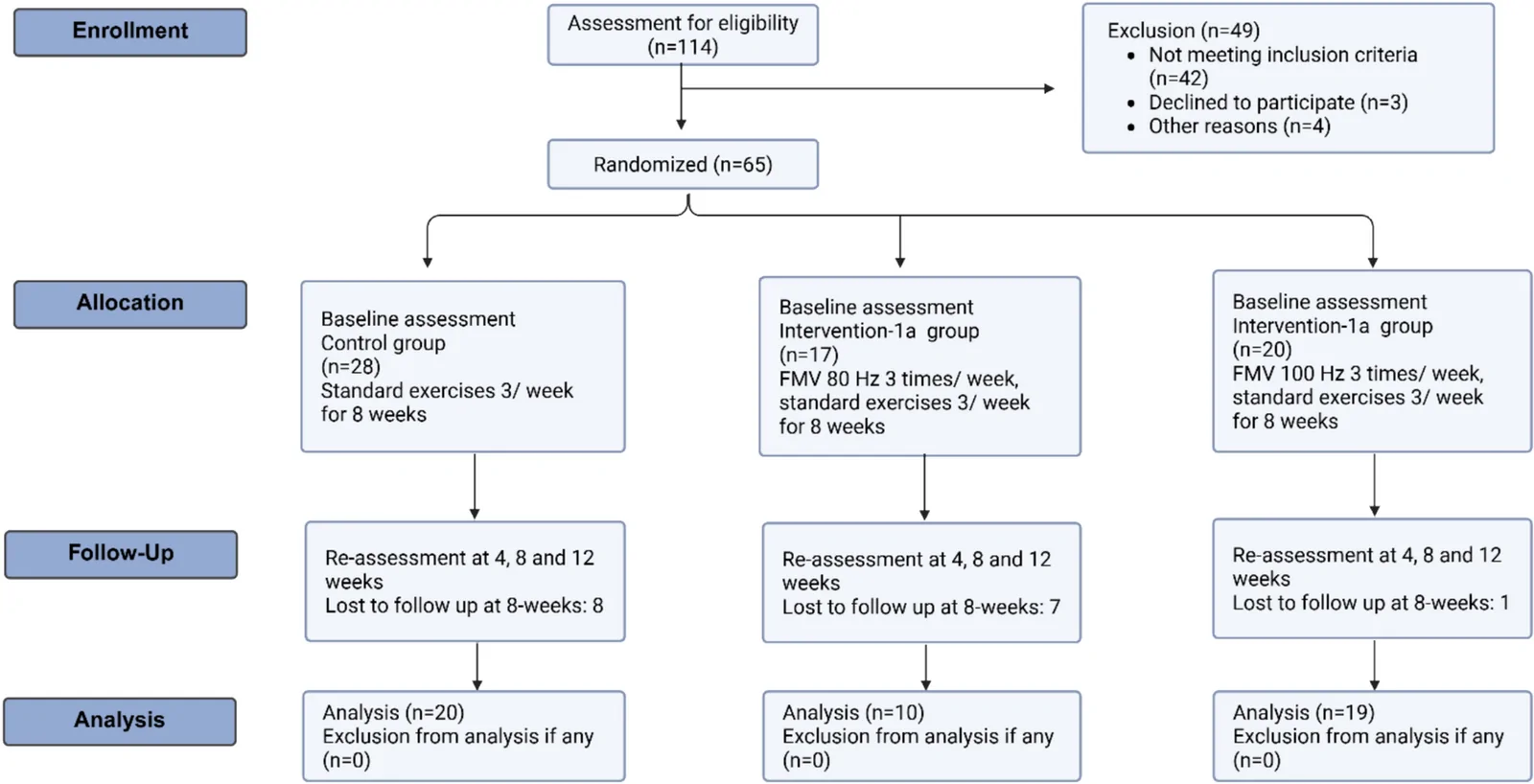
CONSORT diagram for the experiments

A total of 65 participants were recruited for the study; 55 were retained at the 4-week assessment, and 6 were lost to follow-up by the 8-week assessment. Therefore, a total of 49 participants completed the complete 8-week intervention treatment. Modified Ashworth scale scores of the participants were 1 (12), 1 + (18), 2 (25), and 3 (10). The gross motor function classification system (GMFCs) of the recruited participants was as follows: 9 subjects in level II, 34 in III, and 22 in level IV. The study was a double blinded randomized controlled trial with a three-group parallel design. The control group consisted of 20 participants, the Intervention-1a (80 Hz) group had 10, and the Intervention-1b (100 Hz) group had 19 participants. The 12-week assessments to check retention effects were conducted on half (27 participants) of the study population with the following population distribution: 9, 8 and 11 participants in the control, Intervention-1a, and Intervention-1b groups, respectively. The CONSORT diagram for the study detailing the data collection procedures is visualized in Fig. 1. Of the participants with volitional control over their lower limbs, all were right leg dominant. The dominant leg was determined by asking the children which leg they would use to kick a football with. The children who were able to ambulate without assistance were also instructed to mimic a football kick.

Data collection took place from March to August 2023 et al. Farabi Special Education Centre and the National Institute of Rehabilitation Medicine, located in Islamabad, Pakistan. The trial was prospectively registered with United States clinical trial registry (clinicaltrials.gov) with trial ID: NCT05751135. Prior to enrolment, a detailed explanation of the experimental procedures was provided to the children and their parents. Written informed consent was obtained from the parents of the participating children. Verbal assent from the children was also recorded, with those able to speak or nod willingly proceeding with the study. The study was carried out in compliance with the Declaration of Helsinki's ethical standards and with approval from the ethical committee of Riphah International University, Islamabad, Pakistan (REC-01289).

### Procedures

#### Intervention protocol

The FMV intervention was delivered to participants using a custom-built device, reported earlier [44]. The population was divided into three groups: (1) Intervention-1a group received FMV at a frequency of 80 Hz, (2) Intervention-1b group received FMV at 100 Hz. The amplitude of the vibrations was set at 0.5 mm, (3) The control group did not receive any FMV intervention, while all three groups received physical therapy (details below). Participants in the FMV groups were blinded to the specific frequency of vibration they received. However, blinding was not possible for the control group, as the administration of vibration involved the use of a physical device. Stimulation frequencies were chosen based on previous evidence of their effectiveness [20].

**Fig. 2:**
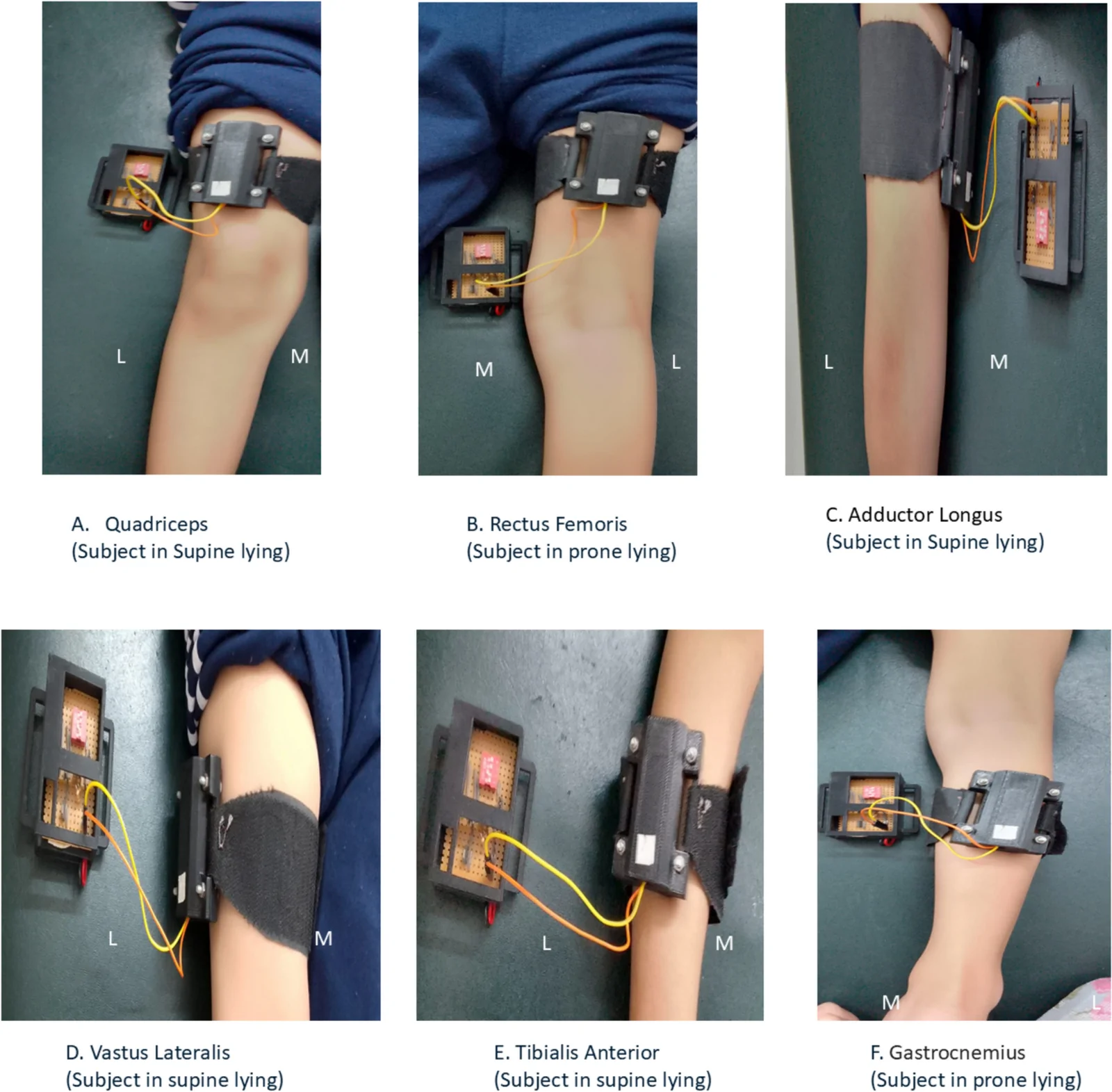
FMV device and the location of the delivery of FMV intervention to the lower limb muscles

The FMV intervention was delivered bilaterally to six lower-limb muscles, four in the thigh: the biceps femoris, vastus lateralis, adductor longus, rectus femoris and two in the distal lower-limb: gastrocnemius, and tibialis anterior (TA) muscles. These muscles were selected because they are commonly associated with spasticity, weakness, impaired selective motor control, and abnormal gait mechanics in children with spastic diplegic cerebral palsy [45–48]. In particular, the adductors and gastrocnemius contribute substantially to scissoring gait and equinus deformity, while tibialis anterior weakness is associated with impaired dorsiflexion and foot clearance during gait. FMV was delivered with participants positioned supine for all muscles except the biceps femoris and gastrocnemius, which were stimulated in the prone position. The right leg was treated first, followed by the left leg. Figure 2 illustrates the FMV device and the placement locations used for vibration delivery.

Vibration stimulus was applied for 30 s, repeated 3 times (90 s) with 10-s intervals. This cycle was repeated 3 times. Each muscle was stimulated for 4.5 min in one session (making a total of 30 min of FMV for the whole limb). FMV was given 3 times a week for 8 weeks. Before the intervention, participants were instructed to lie down on a floor mat. The FMV device was positioned on the belly of the targeted muscle and securely strapped with Velcro straps to minimize lateral movement. The intervention lasted for 8 weeks, with three sessions administered per week (refer to Fig. 4). To ensure consistency, a single physiotherapist conducted all FMV sessions, minimizing intra-participant variability in device placement and muscle tautness.

#### Wearable FMV intervention device

The FMV device was custom-designed [44]. Briefly, it consisted of an eccentric rotating mass motor (Precision Microdrives^©^ 302–102) to generate vibrations regulated by a custom-built electronic circuit to produce frequencies of 80 Hz and 100 Hz. The motor was housed within a 3D-printed casing, and Velcro straps were incorporated to secure the device around the targeted muscles. Additionally, a pressure sensor (Flexiforce^©^ A-201) was integrated into the device to maintain a consistent pressure of 1N during vibration delivery. The device underwent extensive laboratory testing to confirm the accuracy and reliability of its vibration parameters (frequencies and amplitudes. More detailed information about device construction and its operation is presented here [28] in our previous research. See Fig. 2 for a visual representation of device use for the intervention delivery.

#### Physical therapy

All participants received standardized physical therapy interventions in addition to their group allocation. In the intervention groups, focal muscle vibration (FMV) was administered immediately prior to physical therapy to leverage its short-term neuromodulatory effects. FMV is known to enhance proprioceptive input by activating muscle spindle Ia afferents, leading to transient increases in spinal and corticospinal excitability. Delivering FMV prior to active therapy may therefore facilitate a more responsive neuromuscular state, improving motor unit recruitment and responsiveness during subsequent exercises [49]. Evidence suggests that combining vibration-based interventions with motor training can augment neurophysiological and functional outcomes. For instance, repeated muscle vibration applied in conjunction with rehabilitation has been shown to induce sustained increases in cortical excitability and enhance motor recovery in neurological populations [49]. Although these findings are primarily derived from stroke cohorts, similar mechanisms of sensorimotor plasticity are thought to underlie motor impairments in cerebral palsy. Therefore, administering FMV prior to physiotherapy in the present study was intended to optimize the sensorimotor state and enhance the effectiveness of the therapeutic exercises.

Therapy sessions were held three times per week for 8 weeks, lasting approximately 40–60 min each. The rehabilitation program consisted of positioning, stretching, strengthening, and gait training, targeting motor impairments commonly observed in children with spastic diplegic cerebral palsy. These were consistent with previous literature [50, 51] and recommended dosing for physiotherapy interventions in children with cerebral palsy [52]. Details of therapy were as follows:

*Positioning and postural training:* Participants were seated on a block with hips abducted, feet flat on the ground, and the trunk maintained in an upright position to minimize posterior pelvic tilt and promote optimal alignment. Following the exercise session, participants were encouraged to stand against a wall with their legs abducted and externally rotated for 15 min, to improve weight-bearing symmetry and postural control. Parents were instructed to continue these positioning strategies at home to reinforce carryover effects. This is consistent with previous literature [52].

*Stretching protocol:* Passive stretching was applied to spastic muscle groups. Each stretch was held for 20 s, with 5 repetitions per muscle group, at an intensity just below mild discomfort. This approach is consistent with commonly recommended stretching protocols for spasticity management in cerebral palsy [51, 52].

*Strengthening exercises:* Strengthening exercises were performed in 3 sets of 10 repetitions, with 2 min of rest between sets to minimize fatigue. Exercise intensity was individualized using a 10-repetition maximum (10 RM) approach, ensuring that the resistance level corresponded to the maximum load the participant could lift for 10 repetitions with proper form. This method allowed for controlled and progressive overload tailored to each participant’s functional capacity. This is consistent with previous literature [51, 52].

*Gait training:* Gait training was incorporated as a functional component of therapy, focusing on improving walking patterns, balance, and coordination. Activities included assisted ambulation, weight-shifting exercises, and step training, adapted to each participant’s GMFCS level.

*Progression criteria:* Progression of strengthening exercises was guided by periodic reassessment of the participant’s 10 RM. When participants completed the prescribed repetitions with relative ease and proper technique, resistance was increased accordingly to maintain the target intensity. Functional progression was also implemented by advancing from assisted to more independent movements.

#### Electromyography (EMG) assessments

Electromyography (EMG) data were collected from all participants to evaluate the effect of FMV on underlying muscle activation and its influence on co-contraction in children with CP. Assessments were conducted at baseline (prior to the start of interventions), and at 4-, 8-, and 12-week follow-up after the intervention began. The 12-week follow-up was specifically designed to examine the retention effects of the intervention after the 8-week dose had concluded and was performed on half of the study population.

**Fig. 3:**
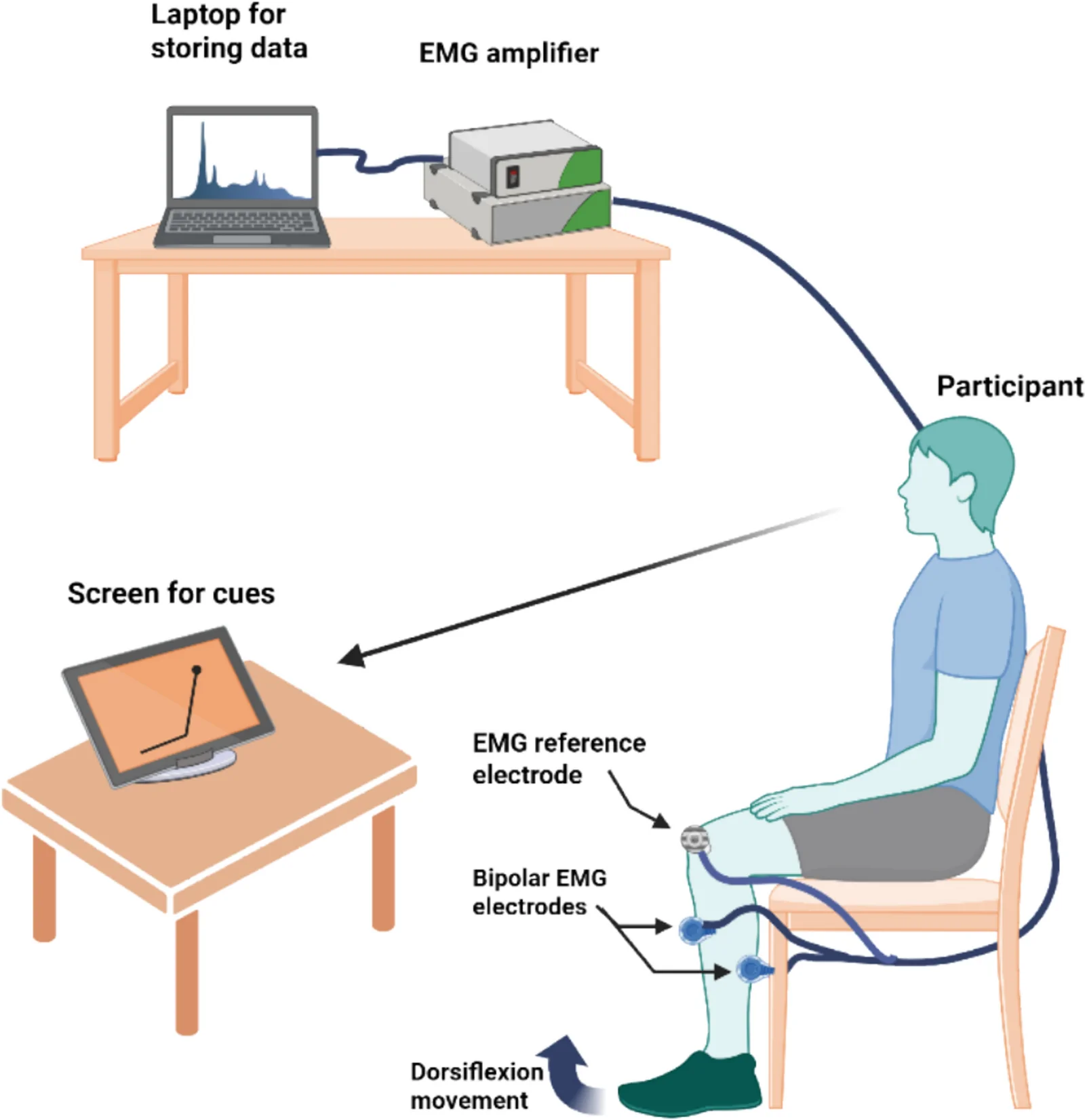
Experimental setup for EMG data collection

**Fig. 4:**
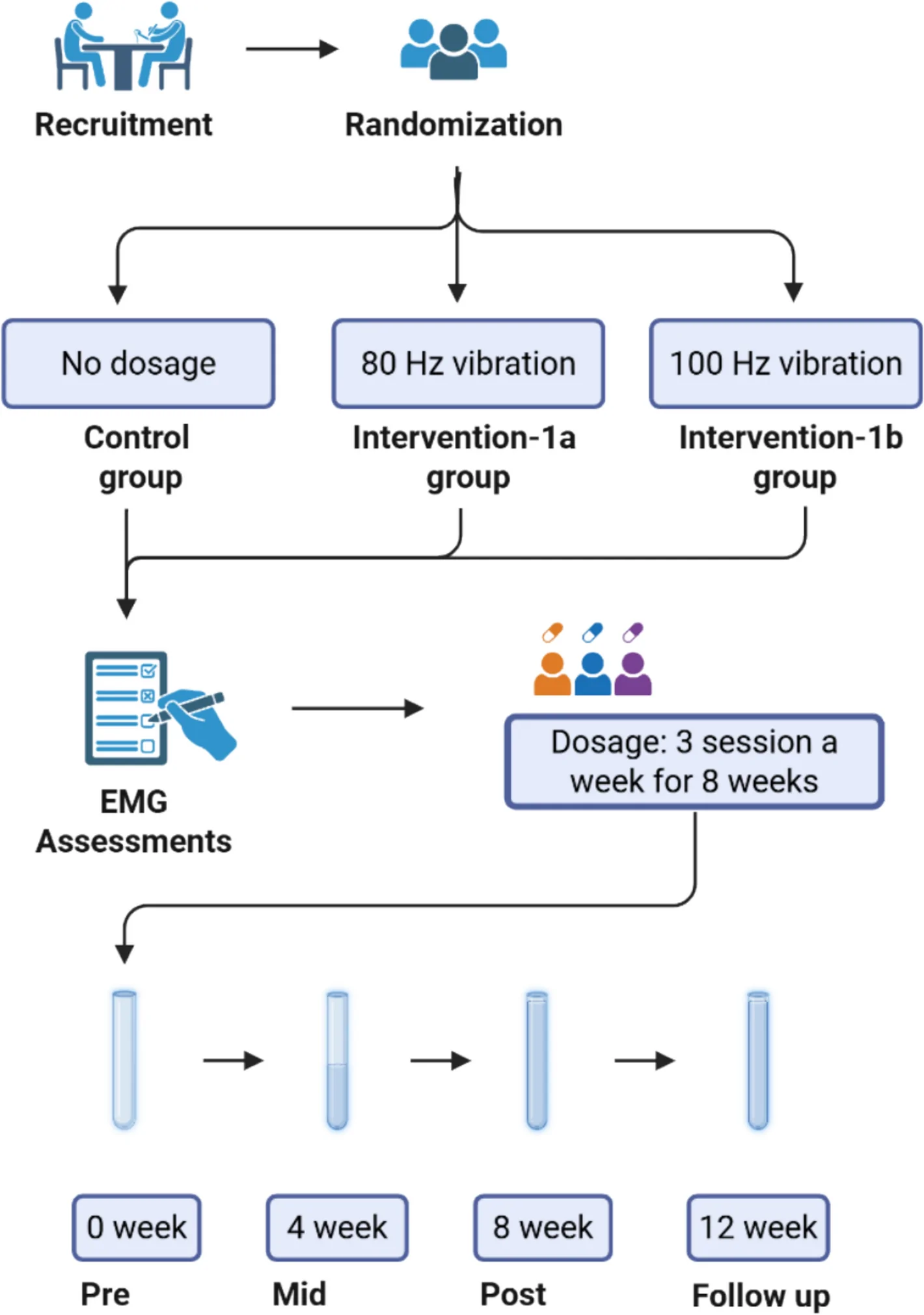
Experimental protocol design for the study

To analyse muscle activations and co-contraction effects, a dorsiflexion (DF) task was employed. Each participant performed 30 DF in response to a visual cue displayed on a smartphone tablet. The start of each DF was timestamped and synchronized with the recorded electromyography (EMG) data. Each DF task lasted 2 s, with a 4-s window (2 s before and 2 s during the task) used for analysis. EMG signals were recorded from the TA and Soleus (Sol) muscles using bipolar electrodes (DelSys, Inc.; model: DE 2.1) with a 1 cm inter-electrode distance connected to the Bagnoli EMG data acquisition system from Delsys (DelSys, Inc.; model: Bagnoli-4). A reference electrode was placed on the knee of each participant. The SENIAM guidelines were followed for electrode placement and data recording [53]. Prior to data collection, the skin was cleaned with isopropyl alcohol or shaved if necessary to ensure optimal signal quality. The experimental setup design is presented in Fig. 3, and the detailed protocol design is shown in Fig. 4.

### Data processing

#### EMG features

EMG data was detrended, rectified, and band-pass filtered to remove noise using a Butterworth filter of 6th order (20–450 Hz). 50 Hz line noise and its harmonics at 100 and 150 Hz were removed using a notch Butterworth filter of the same order, the bandwidth of the filter was set at 1 Hz. All the 30 DF EMG epochs were averaged for each participant for the left and right leg separately. These individual averages were then pooled across the three intervention groups (Intervention-1a (80 Hz), Intervention-1b (100 Hz) and control) to compute EMG features for comparative analysis. All data was processed in MATLAB.

To understand the effects of FMV on the muscular performance EMG signals were analysed and their Root Mean Square (RMS) values were calculated and compared across the study groups (Intervention-1a (80 Hz), Intervention-1b (100 Hz) and control) and assessments (baseline, 4-, 8-, 12-weeks). We also analysed the effect of vibrations on fatigue in the study population, as prolonged use of vibration may affect the fatigue characteristics. We checked how the fatigue behaved over time (baseline, 4-, 8-, 12-weeks). To assess the development of fatigue over time, median frequencies (MDF) of the EMG signal were calculated and compared for the study groups. These features (RMS and MDF) were calculated using source code from [54].

Fatigue was also quantified using a combined frequency-amplitude fatigue index (FI) derived from surface EMG that integrates the spectral and amplitude components of the motor activation pertaining to fatigue:

1 $$FI=\frac{MDF}{RMS}$$

A decrease in the fatigue index corresponds to an increase in muscle fatigue, analogous to classical EMG median-frequency metrics, but with the added advantage of incorporating amplitude-related compensatory mechanisms. This dual-domain approach makes the Fatigue Index more sensitive to fatigue development than frequency-only measures.

To study how FMV affects muscle synergy we calculated co-contraction between the TA and Sol muscles. We examined the behaviour of co-contraction over time, specifically at baseline and at 4-, 8-, and 12-week assessments, across all three study groups, to investigate how FMV might influence contraction patterns.

The co-contraction was measured through the co-contraction index (CI) [55], which is calculated as follows:

2 $$CI=\frac{2{I}_{ant}}{ {I}_{tot}} \times 100$$

where *‘I*_*ant*_*’* and *‘I*_*tot*_*’* represent the area under the EMG graph for the antagonist (TA) muscle and the common area for both muscles, respectively. A CI value of 100% represents total co-contraction, while a value of 0% represents pure agonist activation.

#### Statistical analysis

To analyze the effects of interventions on EMG features (RMS, MDF, FI and CI) values, individual linear mixed-effects model (LMM) were employed, with Group (Control, Intervention-1a (80 Hz), Intervention-1b (100 Hz)) as a fixed between-subjects factor, Time (0, 4, 8, 12 weeks) as a fixed within-subjects factor, and a random intercept for subjects to account for repeated measurements. This approach generalizes the classical two-way repeated measures ANOVA while accommodating missing data and unequal group sizes. Post-hoc pairwise comparisons with Bonferroni correction were conducted for significant interactions.

Assumptions were verified as follows: homogeneity of variance via Levene’s test. For violations, robust LMMs with Kenward-Roger approximation were applied. Post-hoc comparisons used Bonferroni-adjusted *p*-values. Analyses were performed in MATLAB (R2023a, MathWorks) using restricted maximum likelihood (REML) estimation.

## Results

Results for the effect of FMV intervention on muscle activation (EMG root mean square), muscle fatigue (EMG median frequency) and muscle co-contraction are presented below.

### EMG root mean square (RMS)

This study investigated the effects of two interventions, 1) FMV Intervention-1a (at 80 Hz) and 2) FMV Intervention-1b (at 100 Hz), versus a Control group on normalized EMG RMS amplitudes of the TA and Sol muscles bilaterally across four timepoints: baseline, 4, 8, and 12 weeks.

**Fig. 5:**
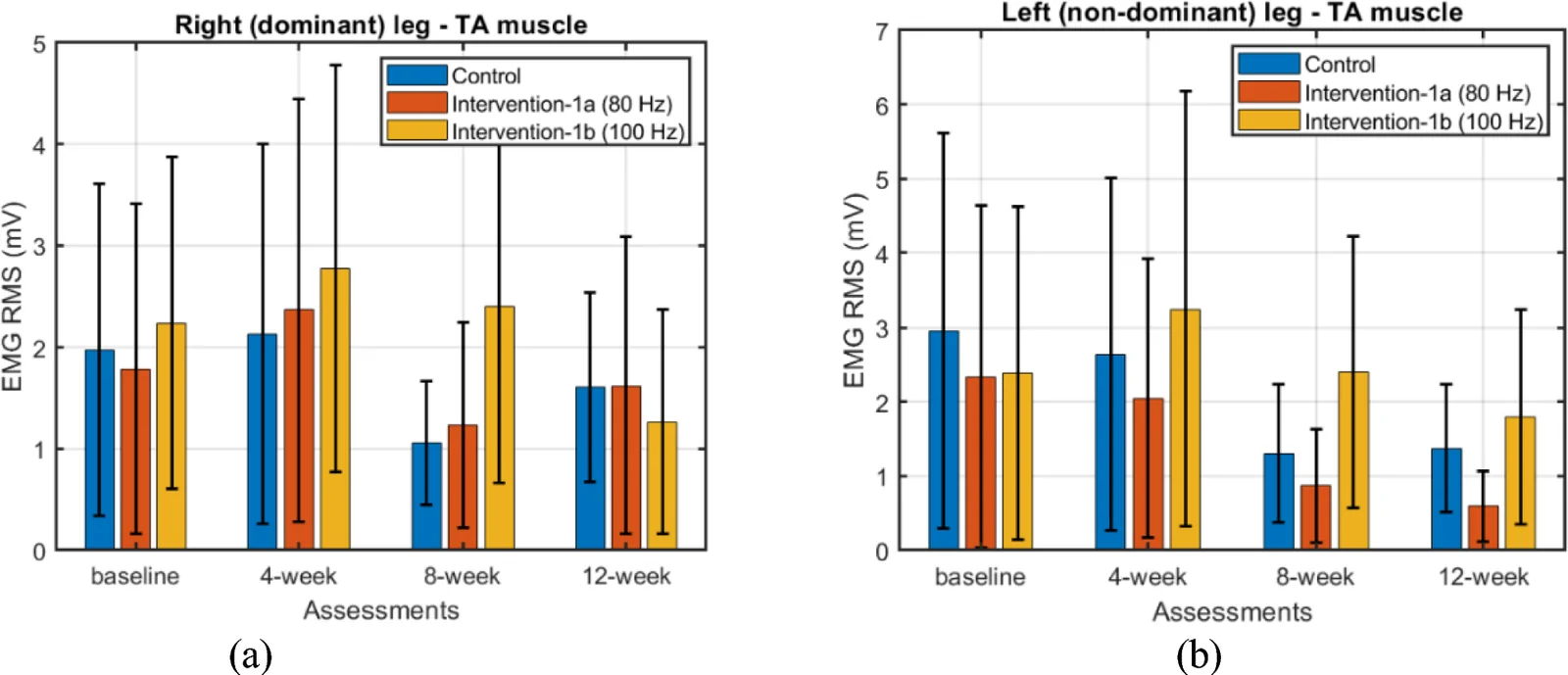
Mean and standard deviation EMG RMS values of TA muscle for the study participants across the intervention groups and the assessment timepoints, for the **a** right and **b** left leg DF tasks

For the right TA (dominant leg) muscle (Fig. 5a), a general trend was observed: mean EMG RMS amplitudes increased at 4 weeks relative to baseline, followed by progressive declines at 8 and 12 weeks. The Intervention-1a group (80 Hz) demonstrated lower baseline amplitudes than controls, increased to exceed control levels by 4 weeks, maintained this elevation at 8 weeks, and returned to control-equivalent values by 12 weeks. In contrast, the Intervention-1b group (100 Hz) exhibited slightly higher baseline amplitudes than controls, further increased at 4 and 8 weeks, but decreased below control levels by 12 weeks. The left TA (non-dominant leg) muscle (Fig. 5b) exhibited analogous trends with attenuated magnitude. The Intervention-1a group (80 Hz) consistently remained below control levels across all time points, suggesting limited efficacy of 80 Hz vibration on the non-dominant limb. Conversely, the Intervention-1b group (100 Hz) started below controls at baseline, increased at 4 and 8 weeks, and declined at 12 weeks while remaining above controls, indicating the potential benefits of 100 Hz vibration for non-dominant limb muscle activation.

**Fig. 6:**
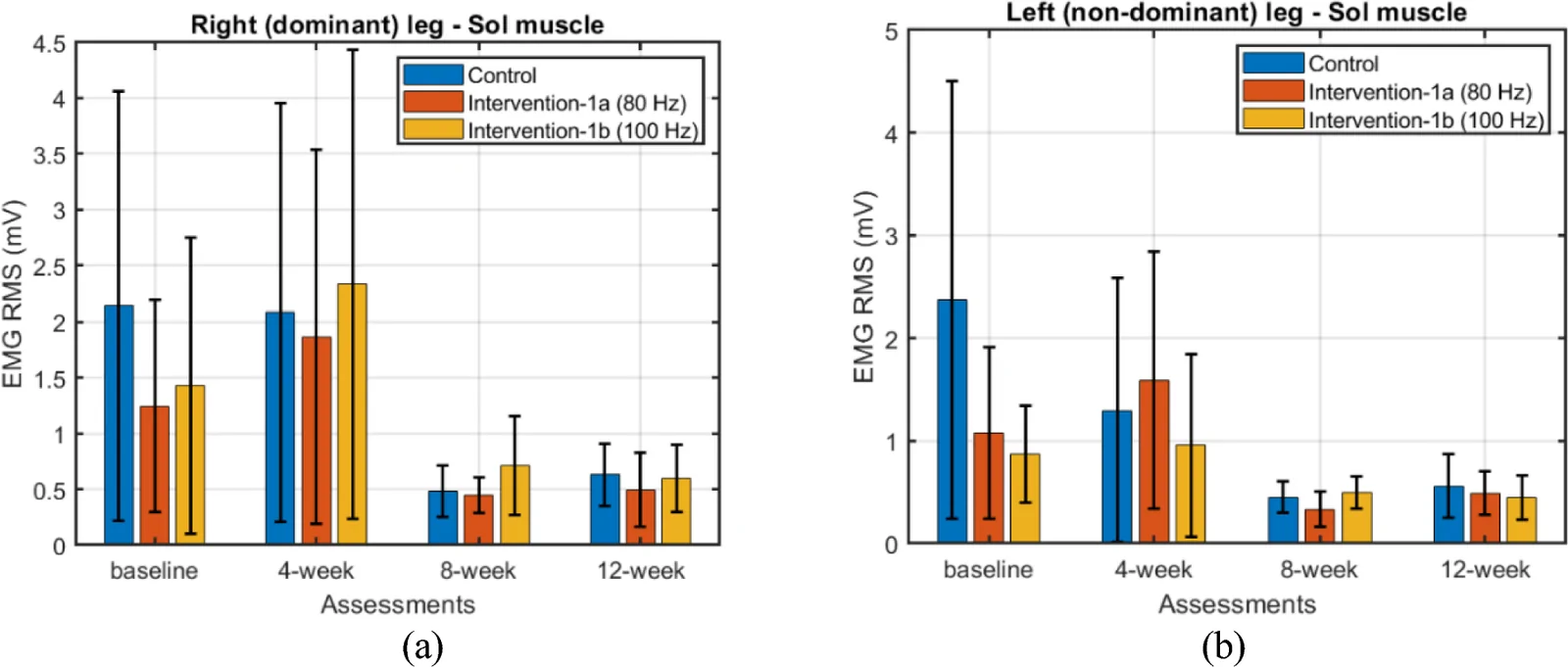
Mean and standard deviation EMG RMS values of Sol muscle for the study participants across the intervention groups and the assessment timepoints, for the **a** right and **b** left leg DF tasks

Trends in Sol mirrored those of their agonist TA counterparts, albeit with reduced amplitude magnitudes. For the right (dominant) Sol (Fig. 6a), both intervention groups began below controls at baseline. By 4 weeks, Intervention-1a (80 Hz) approached control levels, while Intervention-1b (100 Hz) slightly exceeded them. At 8 weeks, Intervention-1a (80 Hz) matched controls, whereas Intervention-1b (100 Hz) surpassed them. By 12 weeks, Intervention-1a (80 Hz) declined below controls, and Intervention-1b (100 Hz) returned to control-equivalent values. In the left (non-dominant) Sol (Fig. 6b), effects were less pronounced. Both intervention groups started below the controls at baseline. At 4 weeks, Intervention-1a (80 Hz) transiently rose above controls, while Intervention-1b (100 Hz) approached control levels. By 8 weeks, Intervention-1a (80 Hz) dipped below controls, and Intervention-1b (100 Hz) marginally exceeded them. At 12 weeks, both groups registered slightly below control levels.

These findings underscore divergent time-dependent effects of vibration frequencies (80 Hz vs. 100 Hz) on neuromuscular activation, with sustained benefits observed for 100 Hz (Intervention-1b) in the non-dominant TA and transient efficacy in other muscle groups.

The statistical analysis revealed the following indications for the experimental findings:

*Right (dominant) leg TA:* A LMM revealed no significant main effects of *Group* (*F*(2163.45) = 2.87, *p* = 0.060) or *Time* (*F*(3149.16) = 1.86, *p* = 0.139), and no *Group* × *Time* interaction (*F*(6177.73) = 1.00, *p* = 0.429). However, post-hoc analysis at 8 weeks showed a significant between-group difference (*p* = 0.003), with Intervention-1b (100 Hz) exhibiting higher EMG RMS than both Control (*p* = 0.003) and Intervention-1a (80 Hz) (*p* = 0.045).

*Left (non-dominant) leg TA:* Significant main effects were observed for *Group* (*F*(2192.36) = 5.84, *p* = 0.003) and *Time* (*F*(3136.56) = 4.03, *p* = 0.009), but no *Group* × *Time* interaction (*F*(6155.98) = 0.96, *p* = 0.455). Post-hoc tests at 4 and 8 weeks demonstrated that Intervention-1b (100 Hz) had significantly higher EMG RMS than both Control (4 weeks: *p* = 0.027; 8 weeks: *p* = 0.027) and Intervention-1a (80 Hz) (4 weeks: *p* = 0.045; 8 weeks: *p* = 0.008).

*Right (dominant) leg Sol:* A significant main effect of *Time* (*F*(3186) = 5.81, *p* = 0.001) was found, but no effects for *Group* (*F*(2186) = 1.15, *p* = 0.320) or *Group* × *Time* (*F*(6186) = 0.82, *p* = 0.559). Post-hoc analysis at 8 weeks showed a marginal between-group effect (*p* = 0.024), but no pairwise differences survived correction. Within-group analyses revealed significant temporal changes in all groups (Control: *p* = 0.046; Intervention-1a (80 Hz): *p* = 0.022; Intervention-1b (100 Hz): *p* = 0.017), with Intervention-1b showing a notable Week 2 vs. Week 3 difference (*p* = 0.035, uncorrected).

*Left (non-dominant) leg Sol:* A significant *Time* effect (*F*(3109.79) = 5.73, *p* = 0.001) was observed, but no *Group* (*F*(2132.61) = 1.92, *p* = 0.151) or *Group* × *Time* (*F*(6164.77) = 1.59, *p* = 0.152) effects. Post-hoc tests at 8 weeks indicated lower EMG RMS in Intervention-1a (80 Hz) compared to Intervention-1b (80 Hz) (*p* = 0.020). Within-group analyses demonstrated significant temporal changes in all groups (Control: *p* = 0.018; Intervention-1a (80 Hz): *p* = 0.013; Intervention-1b (100 Hz): *p* = 0.005), with Intervention-1b (100 Hz) exhibiting reduced activity at Weeks 8/12 versus baseline (*p* = 0.015, uncorrected).

All the statistical results for EMG RMS are presented in Table 1 for the LMM analyses and Table 2 for the post-hoc analyses. Although several post-hoc comparisons reached statistical significance, the corresponding effect sizes were small (Cohen’s d < 0.25), suggesting that the magnitude of change was modest relative to inter-subject variability.

**Table 1:** Summary of LMM results for all conditions for EMG RMS

Muscle	Leg	Effect	F-value	DF (Num, Den)	*p*-value	η^2^
TA	Right	Group	2.87	2163.45	0.060	0.034
		Time	1.86	3149.16	0.139	0.036
		Group × Time	1.00	6177.73	0.429	0.033
	Left	**Group**	**5.84**	2192.36	**0.003**	0.057
		**Time**	**4.03**	3136.56	**0.009**	0.081
		Group × Time	0.96	6155.98	0.455	0.036
Sol	Right	Group	1.15	2186.00	0.320	0.012
		**Time**	**5.81**	3186.00	**0.001**	0.086
		Group × Time	0.82	6186.00	0.559	0.026
	Left	Group	1.92	2132.61	0.151	0.028
		**Time**	**5.73**	3109.79	**0.001**	0.135
		Group × Time	1.59	6164.77	0.152	0.055

The bold texts represent entries with p-values>0.001

**Table 2:** EMG RMS significant post-hoc comparisons (*p* < 0.05)

Muscle	Leg	Timepoint	Comparison	Diff	CI	*p*-value	Cohen’s d
TA	Right	8 weeks	Control versus Intervention-1b	− **2.1 e−6**	**[− 3.56e−6 –0.59e−6]**	**0.003**	**− **0.25
		8 weeks	Intervention-1a versus 1b	− 1.8e−6	[**− **3.6e−6 **− **0.03e−6]	0.045	**− **0.16
	Left	4 weeks	Control versus Intervention-1b	− 1.26e−6	[**− **2.4e−6 **− **0.11e−06]	0.027	**− **0.19
		4 weeks	Intervention-1a versus 1b	− 1.30e−6	[**− **2.58e−6 **− **0.002e−6]	0.045	**− **0.17
		8 weeks	Control versus Intervention-1b	− 1.76e−6	[**− **3.36e−6 **− **0.16e−6]	0.027	**− **0.19
		8 weeks	Intervention-1a versus 1b	− **2.47e−6**	**[4.4e−6 − 0.53e−6]**	**0.008**	**− **0.23
Sol	Left	8 weeks	Intervention-1a versus 1b	− 0.22e–6	[**− **0.40e−06 **− **0.03e−06]	0.020	**− **0.22

The bold texts represent entries with p-values>0.001

### EMG median frequency (MDF)

**Fig. 7:**
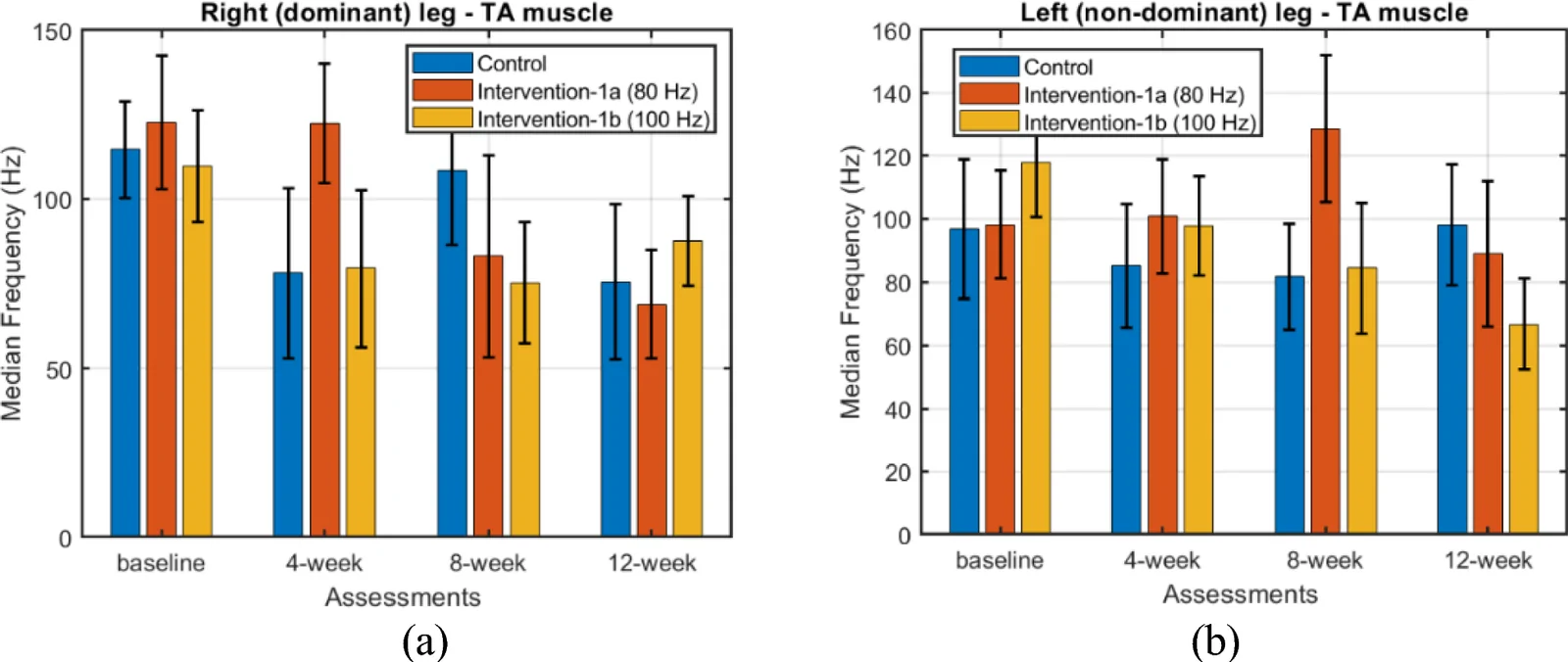
Mean and standard deviation EMG MDF values of TA muscle for the study participants across the intervention groups and the assessment timepoints, for the **a** right and **b** left leg DF tasks

EMG median frequency (MDF) analysis revealed distinct fatigue and activation patterns across intervention groups and muscles. For the right (dominant) leg TA muscle (Fig. 7a), the Intervention-1a (80 Hz) group exhibited marginally higher baseline MDF values than controls, which increased at 4 weeks before declining below control levels by 8 weeks. A modest rebound at 12 weeks restored values to near-control equivalence. In contrast, the Intervention-1b (100 Hz) group demonstrated baseline MDF values slightly below controls, transiently increased at 4 weeks, sharply decreased at 8 weeks, and rebounded above controls by 12 weeks. In the left (non-dominant) leg TA muscle (Fig. 7b), the Intervention-1a (80 Hz) group matched control MDF values at baseline, increased above controls at 4 and 8 weeks, but declined below controls by 12 weeks. The Intervention-1b (100 Hz) group began with a higher baseline MDF than controls, maintained this elevation at 4 weeks, equilibrated with controls at 8 weeks, and dropped below controls by 12 weeks.

**Fig. 8:**
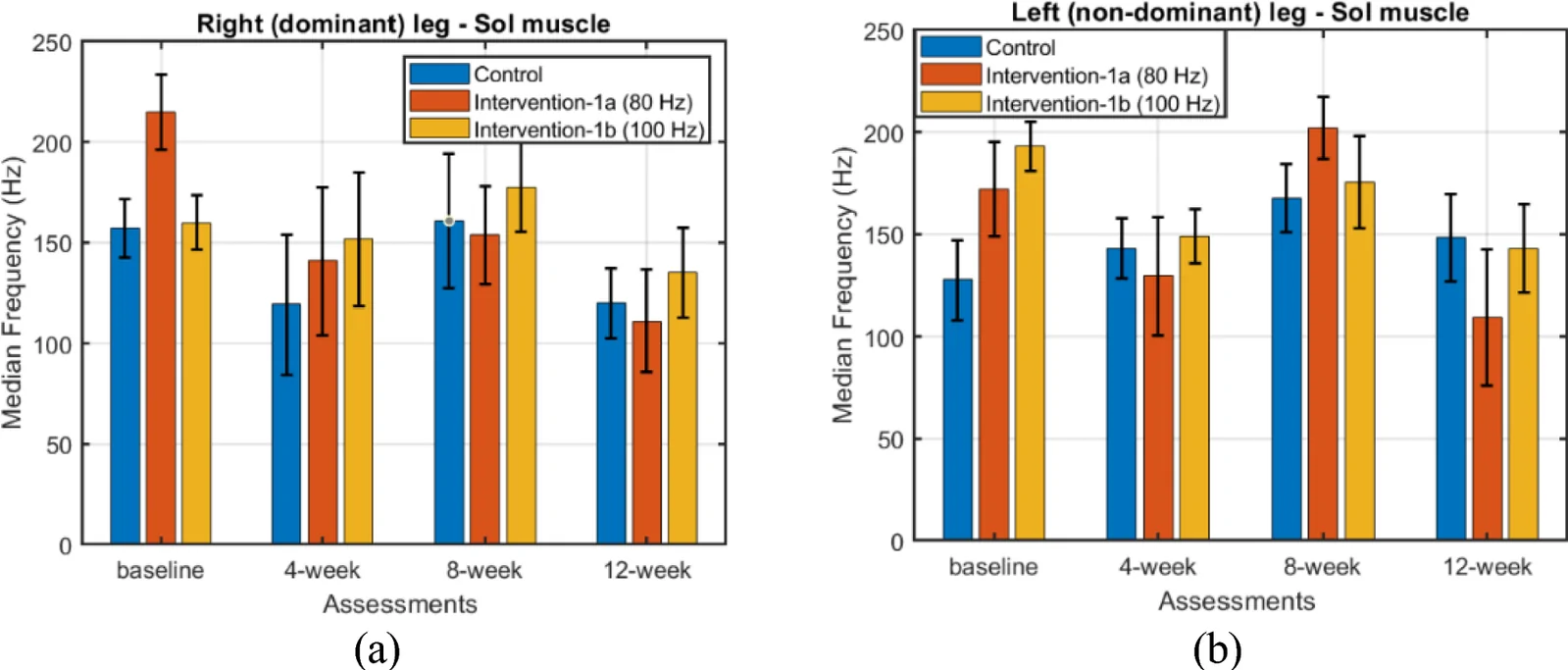
Mean and standard deviation EMG MDF values of Sol muscle for the study participants across the intervention groups and the assessment timepoints, for the **a** right and **b** left leg DF tasks

For the right (dominant) leg Sol muscle (Fig. 8a), the Intervention-1a (80 Hz) group displayed elevated baseline MDF relative to controls, followed by progressive declines at 4, 8, and 12 weeks. Conversely, the Intervention-1b (100 Hz) group matched controls at baseline, increased at 4 weeks, and sustained elevated MDF through 12 weeks. In the left (non-dominant) Sol (Fig. 8b), the Intervention-1a (80 Hz) group fluctuated dynamically: baseline values exceeded controls, dipped below at 4 weeks, rebounded above controls at 8 weeks, and declined below controls by 12 weeks. The Intervention-1b (100 Hz) group, initially higher than controls at baseline, normalized to control levels by 4 weeks and remained stable thereafter.

These MDF trajectories suggest frequency- and muscle-specific fatigue dynamics, with 100 Hz (Intervention-1b) eliciting more sustained activation in the right (dominant) Sol and transient resilience in the TA, while 80 Hz (Intervention-1a) induced progressive fatigue in the right (dominant) Sol and variable responses in the non-dominant limb. It should be noted that MDF, EMG, and fatigue have an inverse relation.

Statistical analysis revealed the following significance of the results:

TA: For both left and right tibialis anterior muscles, the LMM showed no significant group effects (Left: F(2,195.14) = 0.35, *p* = 0.70; Right: F(2,194.09) = 0.38, *p* = 0.68) or *Group* × *Time* interactions (Left: F(6,174.4) = 1.13, *p* = 0.35; Right: F(6,177.83) = 0.91, *p* = 0.49). The right tibialis anterior demonstrated a significant *Time* effect (F(3,152.78) = 3.61, *p* = 0.015), though post-hoc analyses failed to identify specific time points driving this effect. No significant post-hoc differences were found for either leg.

*Sol*: More pronounced effects were observed in the soleus muscles. Both left and right Sol showed significant *Time* effects (Left: F(3,135.43) = 4.32, *p* = 0.006; Right: F(3,148.88) = 4.01, *p* = 0.009), indicating temporal changes in median frequency. For the left (non-dominant) soleus: (1) baseline differences were found between Group 1 (Control) and Group 3 (Intervention-1b, 100Hz) (*p* = 0.020), (2) Intervention-1a (80 Hz) group showed significant *Time* effects (*p* = 0.013), with 8-week vs 12-week differences (*p* = 0.030). The right (dominant) soleus demonstrated significant time effects in the Intervention-1a (80 Hz) group (*p* = 0.030), particularly between 4 and 12 weeks (*p* = 0.049) Tables 3 and 4.

### Fatigue index (FI)

**Fig. 9:**
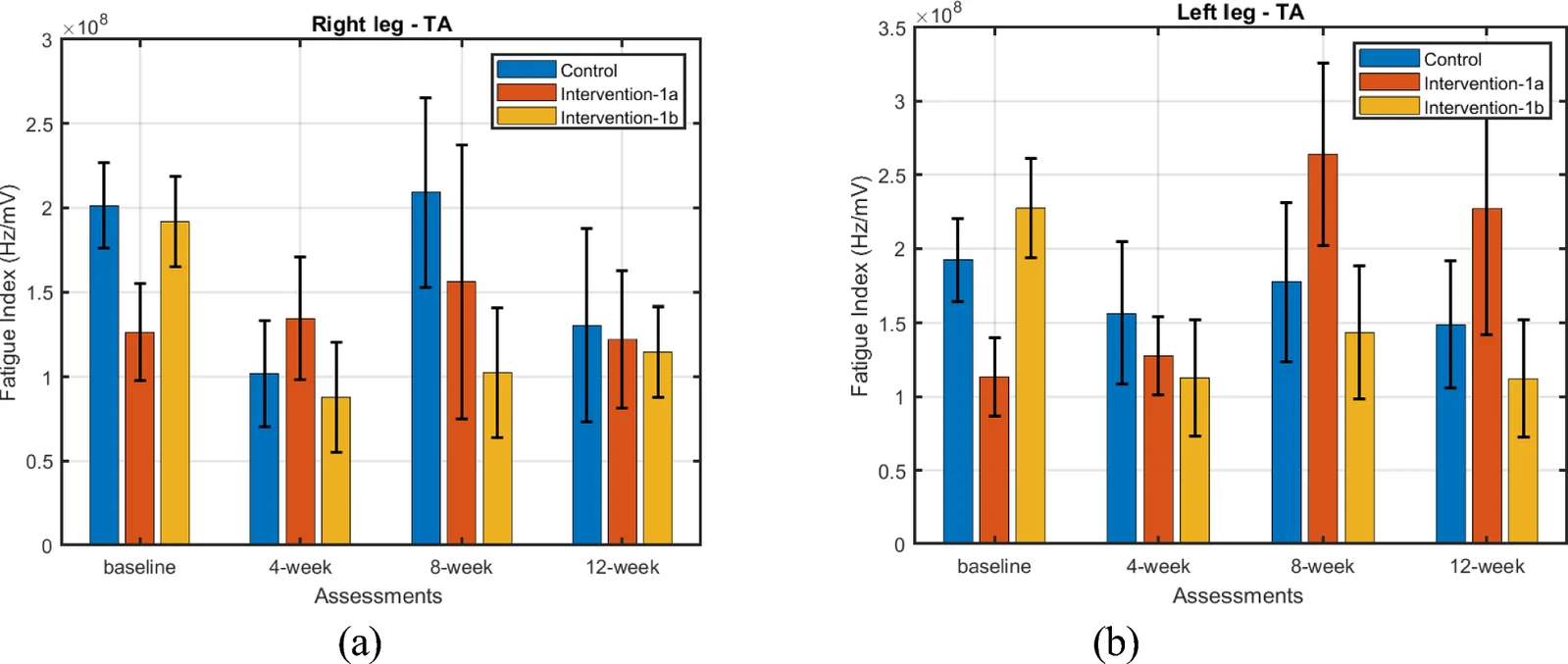
Mean and standard deviation EMG FI values of TA muscle for the study participants across the intervention groups and the assessment timepoints, for the **a** right and **b** left leg DF tasks

**Fig. 10:**
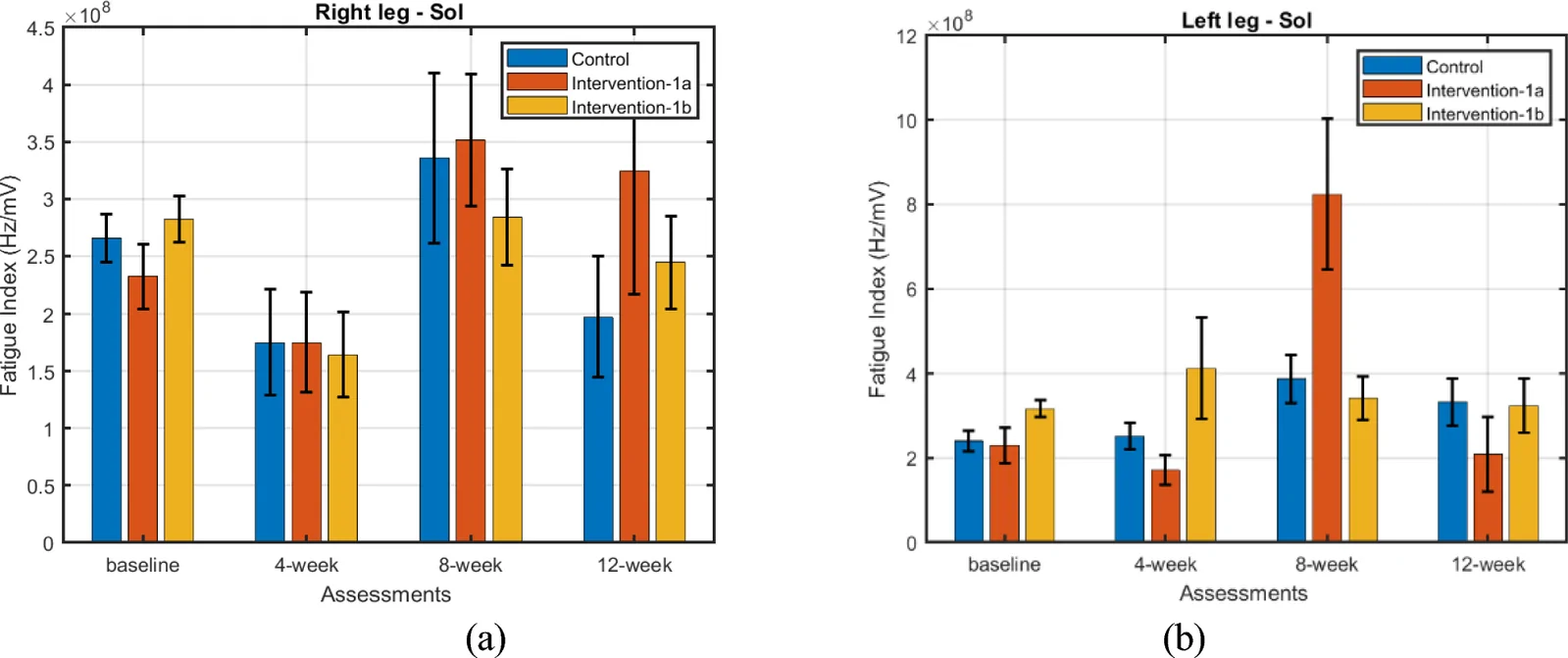
Mean and standard deviation EMG FI values of Sol muscle for the study participants across the intervention groups and the assessment timepoints, for the **a** right and **b** left leg DF tasks

The trends in FI follow a similar pattern to the MDFs for both TA and the left (non-dominant) Sol muscles, except that the control group had higher amplitudes in left TA (see Figs. 9a, b, and 10b). For the right (dominant) leg Sol muscle (Fig. 10a), the Intervention-1a (80 Hz) group displayed lower baseline MDF relative to controls, followed by progressive increases at 4, 8 and 12 weeks. Conversely, the Intervention-1b (100 Hz) group exceeded controls at baseline, decreased at 4 and 8 weeks, and rebounded above baseline at 12 weeks (Table 5).

**Table 3:** Summary of LMM results for all conditions for Median Frequency

Muscle	Leg	Effect	F-value	DF (Num, Den)	*p*-value	η^2^
TA	Left	Group	0.35	2195.14	0.703	0.004
		Time	0.78	3149.62	0.508	0.015
		Group × Time	1.13	6174.40	0.348	0.037
	Right	Group	0.38	2194.09	0.681	0.004
		**Time**	**3.61**	3152.78	0.015	0.066
		Group × Time	0.91	6177.83	0.492	0.03
Sol	Left	Group	1.02	2181.02	0.361	0.011
		**Time**	**4.32**	3135.43	**0.006**	0.087
		Group × Time	1.71	6163.23	0.122	0.059
	Right	Group	0.82	2171.81	0.441	0.009
		**Time**	**4.01**	3148.88	**0.009**	0.075
		Group × Time	0.93	6185.50	0.477	0.029

The bold texts represent entries with p-values>0.001

**Table 4:** Median frequency significant post-hoc comparisons (*p* < 0.05)

Muscle	Leg	Condition	Comparison	Difference	CI	*p*-value	Cohen’s d
Sol	Left	Baseline	Control versus Intervention-1b	**− **65.515	[− 123.092, **− **7.938]	0.020	**− **0.17
		Intervention-1a	8- versus 12-week	92.870	[6.175, 179.568]	0.030	0.19
	Right	Intervention-1a	4- versus 12-week	103.54	[0.1419, 207.166]	0.049	0.16

**Table 5:** Summary of LMM results for all conditions for Frequency Index

Muscle	Leg	Effect	F-value	DF (Num, Den)	*p*-value	η^2^
TA	Left	Group	0.30	2195.53	0.736	0.003
		Time	0.58	3150.04	0.624	0.012
		Group × time	0.89	6174.37	0.498	0.03
	Right	Group	0.35	2184.21	0.702	0.004
		Time	0.73	3156.35	0.530	0.014
		Group × time	0.32	6185.56	0.923	0.01
Sol	Left	Group	0.39	2179.63	0.672	0.004
		**Time**	**5.41**	**3156.43**	**0.001**	0.094
		**Group × time**	**2.99**	**6187.02**	**0.008**	0.088
	Right	Group	0.20	2178.4	0.815	0.002
		Time	2.55	3155.07	0.057	0.047
		Group × time	0.27	6185.73	0.950	0.009

The bold texts represent entries with p-values>0.001

Statistical analysis revealed the following significance of the results:

TA: For either left or right TA, the LMM revealed no significant *Group* effects*, Time* effects or *Group* × *Time* effects. See Table 6.

**Table 6:** Fatigue index (FI) significant post-hoc comparisons (*p* < 0.05)

Muscle	Leg	Condition/ timepoint	Comparison	Difference	CI	*p*-value	Cohen’s d
Sol	Left	8-week	Control vs. Intervention-1a	− 4.36e+8	[− 8.17e+8, − 5.54e+7]	0.019	– 0.19
		8-week	Intervention-1a vs. Intervention-1b	4.81e+8	[1.04e+8, 8.58e+8]	0.008	0.21
	Left	**Intervention-1a**	**baseline vs. 8-week**	− **5.95e+8**	**[**− **9.91e+8, **− **1.97e+8]**	**0.0009**	– 0.27
		**Intervention-1a**	**4-week vs. 8-week**	− **6.53e+8**	**[**− **1.06e+9, **− **2.41e+8]**	**0.0004**	– 0.28
		Intervention-1a	8-week vs. 12-week	6.14e+8	[1.42e+8, 1.086e+9]	0.005	0.22

The bold texts represent entries with p-values>0.001

*Sol*: No significant effects were seen for the right Sol muscle. For the left *Time* effects (F(3156.43) = 5.41, *p* = 0.001) and *Group* × *Time* effects (F(6187.02) = 2.99, *p* = 0.008) were observed. Post-hoc analysis revealed significant interactions at 8 weeks: Control vs intervention-1a (80 Hz) (*p* = 0.019) and intervention-1a (80 Hz) vs intervention-1b (100 Hz) (*p* = 0.008). Significant interactions were also observed for intervention-1a (80 Hz): for baseline vs 8-weeks (*p* = 0.0009), 4-weeks vs 8-weeks (*p* = 0.0004), and 8-weeks vs 12-weeks (*p* = 0.005). Table 6.

Although several post-hoc comparisons reached statistical significance, the corresponding effect sizes for both MDF and FI analysis were small (Cohen’s d < 0.30), suggesting that the magnitude of change was modest relative to inter-subject variability.

### EMG co-contraction index (CCI)

**Fig. 11:**
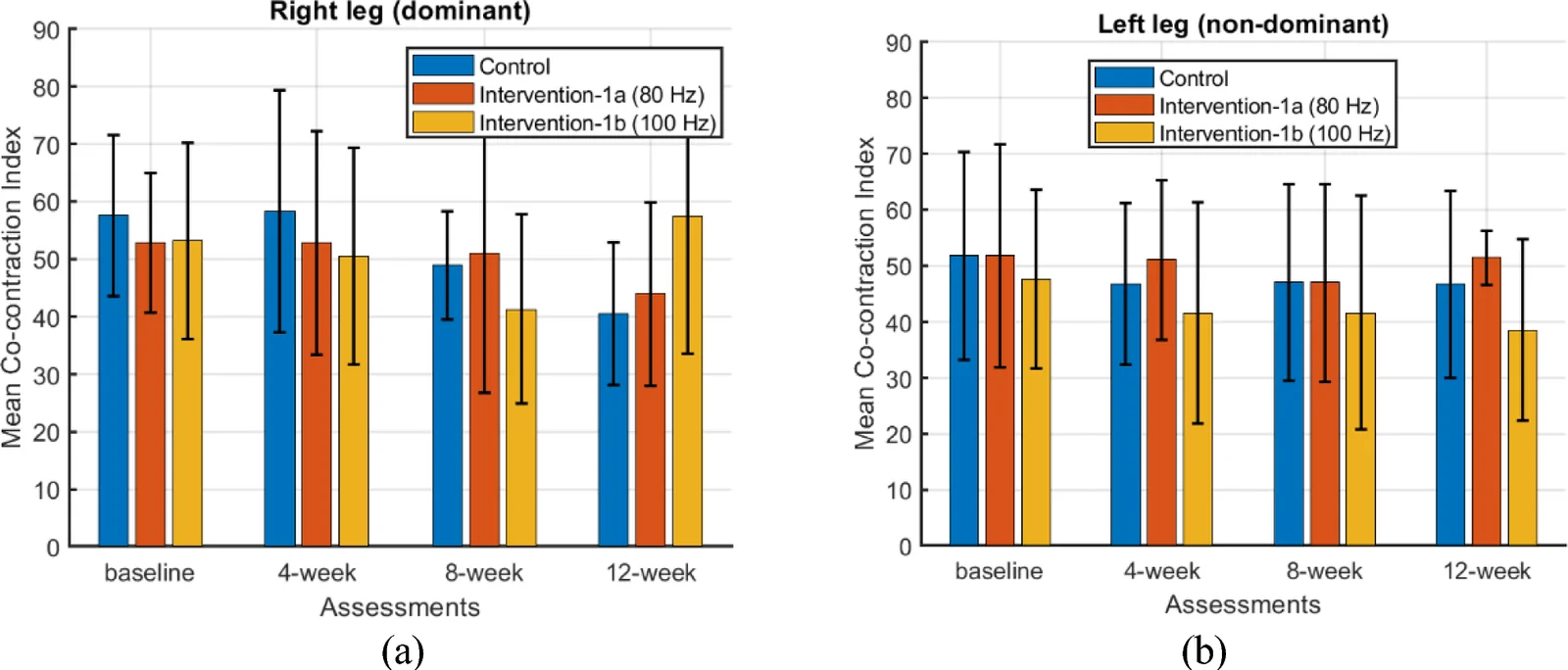
Mean and standard deviation CCI values between TA and Sol muscle across the intervention groups and the assessment timepoints, for the **a** right and **b** left leg DF tasks

CCI analysis revealed distinct temporal and interventional patterns in agonist–antagonist coordination (Fig. 11a–b). For the right (dominant) leg, both intervention groups exhibited lower baseline and 4-week CCI values compared to controls. The Intervention-1b (100 Hz) group remained below Controls until 8 weeks but surpassed them by 12 weeks, whereas the Intervention-1a (80 Hz) group marginally exceeded controls at 8 weeks and stabilized at this elevated level through 12 weeks, indicative of a gradual, vibration-modulated increase in co-contraction activity. In the left (non-dominant) leg, divergent trends emerged. The Intervention-1a (80 Hz) group demonstrated fluctuating CCI dynamics: baseline values matched controls, exceeded them at 4 weeks, settled back to control-level at 8 weeks, and rebounded above controls by 12 weeks; possibly showing an adaptation to intervention at the 8-week point, but an improvement effect in the long-term (12 weeks). Conversely, the Intervention-1b (100 Hz) group exhibited persistently lower CCI values than controls across all time points, suggesting a minimal influence of 100 Hz vibration on co-contraction modulation in the non-dominant limb.

Lower CCI reflects reduced unwanted co-activation (improved control), whereas higher CCI may indicate compensatory stiffness. These findings highlight limb- and frequency-dependent disparities in neuromuscular coordination, with 80 Hz (Intervention-1a) eliciting progressive adaptation in the dominant leg and adaptive non-dominant responses. In comparison, 100 Hz (Intervention-1b) showed delayed dominance-leg efficacy and no detectable effects on non-dominant co-contraction. Low statistical significance suggests interpreting these results with caution.

Right (dominant) leg showed different coordination patterns: a significant main effect of *Time* (F(3155.29) = 3.14, *p* = 0.027) was found, and no group effects (*p* = 0.808) or interaction (*p* = 0.240) effects were found. No post-hoc significant effects were identified. In the left (non-dominant) limb, a significant main effect of *Group* was observed (F (2192.57) = 3.46, *p* = 0.034), indicating co-contraction patterns differences between intervention and control groups. However, post-hoc analyses failed to identify specific group differences. No significant time effects (*p* = 0.256) or *Group* × *Time* interactions (*p* = 0.908) were found. See Table 7.

**Table 7:** Summary of LMM results for all conditions for CCIs

Leg	Effect	F-value	DF (Num, Den)	*p*-value	η^2^
Left	Group	3.46	2192.57	0.034	0.035
	Time	1.37	3152.38	0.256	0.026
	Group × time	0.35	6178.96	0.908	0.012
Right	Group	0.21	2183.48	0.808	0.002
	Time	3.14	3155.29	0.027	0.057
	Group × time	1.34	6186.90	0.240	0.041

## Discussion

The present study investigated the effects of focal muscle vibration (FMV) at two frequencies (80 Hz and 100 Hz) on neuromuscular activation, fatigue, and co-contraction patterns (between agonist–antagonist pair) in children with spastic diplegic cerebral palsy (CP). The findings revealed frequency-dependent, time-varying, and muscle-specific responses across EMG RMS (muscle activation), MDF (fatigue dynamics), and CCI (agonist–antagonist coordination). These results provide critical insights into the therapeutic potential of FMV for modulating motor control in CP while underscoring the complexity of neuromuscular adaptations to vibration interventions. Below, we discuss the implications of these findings in the context of existing literature, clinical relevance, and methodological considerations.

### Muscle activation

The normalized EMG RMS amplitudes demonstrated divergent trends between intervention groups and limbs. For the dominant (right) tibialis anterior (TA), the 100 Hz group (Intervention-1b) exhibited a transient increase in activation at 4 and 8 weeks, followed by a decline to baseline by 12 weeks. In contrast, the 80 Hz (Intervention-1a) group showed sustained activation in the dominant TA but limited efficacy in the non-dominant (left) TA. Similar trends were observed in the soleus (Sol), with 100 Hz eliciting transient activation peaks and 80 Hz showing progressive declines. These findings suggest that higher-frequency vibrations (≥ 100 Hz) may enhance acute neuromuscular recruitment, potentially through increased muscle spindle modulation, while lower frequencies (≤ 80 Hz) may promote longer-term adaptations in muscle tone and stiffness. These observations are supported by earlier research, which suggests that neuromuscular responses are dependent on vibration stimulation frequencies [19, 56].

The statistical analysis reinforced these observations. For the left TA, a significant main effect of *Group* (F(2, 192.36) = 5.84, *p* = 0.003, η^2^ = 0.057) highlighted the superiority of 100 Hz (Intervention-1b) in increasing RMS amplitudes compared to controls and 80 Hz (Intervention-1a), particularly at 4 and 8 weeks. This suggests that 100 Hz may transiently improve muscle activation and/or inhibit spasticity in non-dominant limbs, potentially by modulating reflex pathways or reducing co-contraction. However, the lack of significant *Group* × *Time* interactions in most muscles implies that the interventions’ effects were consistent over time rather than progressively amplifying. Furthermore, the low effect size values (η^2^ < 0.06 and Cohen’s d < 0.20) indicate that the magnitude of change was small.

The transient nature of RMS changes—particularly the return to baseline by 12 weeks—underscores the need for a continual vibration dose to sustain therapeutic gains. The decline in RMS amplitudes at 12 weeks may reflect neural adaptation or habituation to vibration stimuli. Clinically, these results suggest that 100 Hz FMV could be prioritized for short-term muscle activation/ function improvement and/or spasticity management, while 80 Hz may require adjunct therapies (e.g., task-specific training) to prolong its effects.

### Fatigue and sustained activation

Median frequency (MDF) analysis provided insights into fatigue dynamics, with lower MDF values indicating greater fatigue. The 100 Hz (Intervention-1b) group demonstrated resilience in the dominant Sol, sustaining elevated MDF through 12 weeks, while the 80 Hz (Intervention-1a) group exhibited progressive fatigue in the same muscle. Conversely, in the non-dominant TA, 80 Hz initially increased MDF (suggesting reduced fatigue) but declined sharply by 12 weeks. FI displayed similar trends. These frequency- and muscle-specific responses may stem from differences in muscle fiber recruitment: higher frequencies may preferentially activate the underlying fibers at a different rate than lower frequencies, thereby stemming the difference in behaviors [57, 58].

The significant *Time* effects in both Sol muscles (left: F(3, 135.43) = 4.32, *p* = 0.006, η^2^ = 0.087; right: F(3, 148.88) = 4.01, *p* = 0.009, η^2^ = 0.075) indicate that fatigue accumulated universally across groups, likely due to the demanding intervention protocol (three weekly sessions). However, the 100 Hz (Intervention-1b) group’s ability to sustain MDF in the dominant Sol suggests that this frequency may delay fatigue onset in postural muscles critical for gait—a finding directly relevant to CP rehabilitation, where fatigue resistance is a key therapeutic target. The transient MDF rebound in the 100 Hz (Intervention-1b) group’s dominant TA at 12 weeks further implies that intermittent "rest periods" in vibration dosing could potentially enhance performance-related outcomes. Notably, the effect sizes for the Sol muscle indicate small-to-medium changes in both the non-dominant (η^2^ < 0.09; Cohen’s d < 0.20) and dominant legs (η^2^ < 0.08; Cohen’s d < 0.20).

Notably, the lack of significant *Group* × *Time* interactions for MDF suggests that both frequencies induced comparable fatigue trajectories, differing only in magnitude, potentially due to variability in motor unit recruitment thresholds or spasticity severity. Future studies should explore personalized vibration parameters tailored to individual muscle characteristics (e.g., spasticity grade).

In addition to MDF, incorporating the composite fatigue index (FI), which integrates the spectral and amplitude components of the EMG signal, provides a more nuanced understanding of fatigue dynamics under FMV intervention. The FI trends largely paralleled MDF findings, reinforcing the reliability of the observed fatigue patterns while offering additional sensitivity to compensatory neuromuscular strategies. Notably, significant Group × Time (F(6, 187.02) = 2.99, *p* = 0.008, η^2^ = 0.088) interactions in the non-dominant soleus muscle demonstrate that the pattern of fatigue progression differed between groups, even though absolute baseline FI values did not differ. This suggests that the intervention or group-specific condition influenced how fatigue developed over time, rather than initial fatigue levels. Given that lower FI values correspond to greater fatigue, the observed reductions in FI over time for the left soleus represent increasing muscle fatigue, with group-dependent trajectories. Although a trend toward temporal change was observed, the fatigue index of the right soleus did not reach statistical significance. This indicates asymmetric fatigue responses, with significant fatigue development occurring only in the left soleus. The effect sizes support this pattern: the left soleus showed small-to-medium effects (η^2^ ≈ 0.09; Cohen’s d = 0.19–0.28), reflecting modest but measurable changes in fatigue characteristics. In comparison, the right soleus did not exhibit statistically significant changes.

These findings suggest that while MDF reflects general fatigue trends, FI may better capture the interplay between motor unit recruitment and compensatory amplitude modulation, which is especially relevant in populations with impaired motor control such as CP. Therefore, the combined use of FI and MDF provides complementary insights, strengthening the interpretation that FMV induces complex, frequency-specific fatigue adaptations rather than uniform changes in muscle endurance.

### Agonist–antagonist co-contraction

CCI analysis revealed limb- and frequency-dependent disparities in agonist–antagonist coordination, with higher CCI values meaning high co-contraction. In the dominant leg, 100 Hz (Intervention-1b) FMV reduced CCI initially but surpassed control levels by 12 weeks, indicating a delayed shift toward co-activation. In contrast, 80 Hz (Intervention-1a) elicited gradual CCI increases, suggesting progressive adaptation. For the non-dominant leg, 100 Hz had minimal impact on CCI, while 80 Hz induced adaptive patterns, first exceeding control levels, coming back to control levels by 8 weeks (the end of the intervention period), but again surpassed at 12 weeks follow-up; again hinting towards a delayed co-activation. These results show limb specificity in the FMV action and suggest a different rate of adaption for different frequencies.

The significant *Group* effect for the left leg CCI (F(2, 192.57) = 3.46, *p* = 0.034, η^2^ = 0.035) highlights 80 Hz FMV’s ability to reduce co-contraction in non-dominant limbs, potentially by normalizing reciprocal inhibition disrupted in CP. However, the absence of post-hoc differences limits definitive conclusions.

Clinically, these findings advocate for combining FMV with activities that reinforce optimal co-contraction patterns (e.g., balance training). The limb-specific responses also emphasize the need for bilateral assessments in CP rehabilitation, as both limbs in CP are known to show different task completion behavior, showing varying compensatory mechanism [59, 60].

### Mechanistic interpretation

It is important to reconcile these observations with the broader vibration literature. In neurologically intact adults, psychophysical studies of self-motion and proprioceptive perception have reported that frequencies below approximately 80 Hz produce little effect, while frequencies above this threshold differ mainly in the duration of the after-effect rather than in its qualitative character [63]. Our findings are consistent with this view: the 80 Hz and 100 Hz protocols did not produce categorically different responses but rather differed in the magnitude and temporal profile of a shared set of adaptations: 100 Hz favouring larger, earlier activation and fatigue-resistance effects, and 80 Hz favouring slower-developing coordination changes. Several factors may explain why these magnitude- and timing-related differences become functionally detectable in children with spastic diplegic CP when they might be negligible in healthy perceptual paradigms. First, the present outcomes are motor (EMG-derived activation, fatigue and co-contraction) rather than perceptual, and are measured in a pathological substrate. Second, in spastic CP the gain of Ia afferent pathways, reciprocal inhibition and corticospinal drive are altered, so identical afferent input can be integrated differently, and small differences in afferent drive may be non-linearly amplified or differently weighted between spinal and supraspinal levels [57, 65, 66]. Consequently, a frequency difference that yields only a change in effect duration in intact perception may surface as a measurable difference in the magnitude and time course of motor adaptation in CP.

Mechanistically, these frequency-dependent effects are best understood within a plasticity framework. Repeated focal muscle vibration delivers trains of synchronized Ia afferent input that act as a conditioning stimulus, progressively strengthening synaptic efficacy at spinal inter-neuronal and motor-cortical levels and producing LTP-like, use-dependent reorganization that can outlast the stimulus [64, 65, 67]. Within this framework, 80 Hz and 100 Hz are not expected to recruit different circuits, but to drive this common plastic process with differing efficiency and time constants, depending on how faithfully each frequency entrains Ia discharge and how regularly that discharge is patterned. This account is consistent with our time-dependent findings: the transient nature of the RMS and MDF effects, which largely returned toward baseline by the 12-week follow-up, suggests that the relatively modest cumulative dose used here was sufficient to initiate, but not to consolidate, the longer-lasting plastic changes described for higher-dose protocols [64, 65], reinforcing the dose–response argument developed below.

### Study limitations and future directions

While this study provides novel insights, several limitations warrant acknowledgment. First, the attrition rate (16 participants lost by 8 weeks) and uneven group sizes (Control: 19, Intervention-1a: 11) may have reduced statistical power, particularly for interaction effects. Second, the inability to blind the control group (due to the visible absence of vibration devices) introduces potential performance bias. Specifically, participants in the control group may have had lower expectations for improvement, which could have inflated the perceived efficacy of the FMV interventions. However, the use of objective sEMG measures rather than subjective clinical scales helps mitigate the impact of this lack of blinding on the primary data. The most likely direction of this bias is an over-estimation of FMV efficacy: an expectancy (Hawthorne-type) effect in the intervention groups, combined with lower outcome expectations in the unblinded control group, would tend to exaggerate between-group differences. While the use of objective sEMG outcomes attenuates this concern, it does not eliminate it, because effort and engagement during the dorsiflexion task may still be influenced by participant expectations. The absence of a sham condition is therefore a limitation of the present design, and the magnitude of the reported between-group differences should be interpreted with this in mind. Future trials should incorporate a properly designed sham condition. For example, a visually identical, non-vibrating device applied with matched contact pressure, together with an acoustic stimulus that mimics motor noise, so that tactile, visual and auditory cues are equated across groups while the vibratory component alone is manipulated. Such a design would allow expectancy effects to be dissociated from the genuine neuromuscular effects of vibration and would substantially strengthen causal inference. Third, the 12-week follow-up assessed only half the cohort, limiting generalizability of retention effects. Additionally, focusing on lower-limb muscles excludes potential systemic or upper-limb effects of FMV. Future work should explore the relationship between vibration in the upper and lower limbs and its impact on functional tasks (e.g., walking, stair climbing). Using a single physiotherapist for all sessions minimized intra-participant variability but may limit reproducibility across clinicians. Further, FMV dosage of 1.5 min/per muscle and 6 min/session in total, could be considered lower to induce meaningful functional changes. For example, earlier FMV studies in stroke population, which have reported significant benefits in spasticity reduction and functional improvement, have delivered a 10 min/muscle/visit FMV dose [35, 46]. Future studies with larger FMV dosage (10 min or more/muscle/visit) in the CP population could further explain dose–response mechanisms. Furthermore, we acknowledge that our final sample size (n = 65) fell short of the projected power analysis requirement (n = 96). While the results demonstrate statistical significance in several key metrics, the study may be underpowered to detect smaller effect sizes or long-term interactions. Future large-scale multi-center trials are required to validate these findings. Overall, the effect sizes were small to medium (η^2^ < 0.10; Cohen’s d < 0.30), indicating that the observed effects, while statistically significant, were modest and may reflect the low-dose intervention delivered to a heterogeneous and underpowered sample.

The frequency-specific effects of FMV highlight its potential as a precision therapy tool in CP management. Clinically, these findings suggest distinct applications for different vibration parameters: 100 Hz FMV appears optimal for short-term spasticity reduction and delaying fatigue in dominant limbs, offering immediate therapeutic benefits. In contrast, 80 Hz FMV, with its progressive activation patterns, may synergize effectively with strength training programs to enhance neuromuscular adaptation over time. Furthermore, combining protocols, such as alternating frequencies, could balance acute relief with sustained improvements, addressing both immediate and long-term rehabilitation goals. To advance these applications, future research should prioritize three key areas. First, extending intervention durations beyond 8 weeks and defining optimal maintenance dosing regimens could clarify FMV’s long-term effects and clinical scalability. Second, mechanistic studies using neuroimaging (e.g., functional magnetic resonance imaging (fMRI)) and neurophysiological tools (e.g., transcranial magnetic stimulations (TMS), electroencephalography (EEG)) are critical to unravelling the neural mechanisms underlying frequency-specific responses, such as cortical reorganization or spinal reflex modulation. Finally, developing personalized FMV protocols tailored to individual factors, including spasticity severity, motor function, or limb dominance, may maximize therapeutic precision and outcomes. Together, these insights could refine FMV as a targeted, adaptable intervention in CP care.

An important consideration when interpreting these findings is the relatively modest FMV dosage used in the present study (1.5 min per muscle, ~ 6 min per limb per session), which is lower than that reported in several prior neurorehabilitation studies. For instance, studies in stroke and neurological populations have commonly employed longer stimulation durations (e.g., ≥ 10 min per muscle per session) [36, 37] and have reported more sustained reductions in spasticity and improvements in functional outcomes. This discrepancy suggests a potential dose–response relationship, where higher cumulative vibration exposure may induce stronger or longer-lasting neuromuscular adaptations, possibly through enhanced afferent drive and greater cortical plasticity. The transient nature of the observed effects at 12 weeks in our study further supports the need for optimized dosing strategies, including longer session durations, increased weekly frequency, or maintenance protocols to sustain benefits. Future research should systematically investigate FMV dose–response characteristics by manipulating duration, intensity, and frequency in a controlled manner to identify optimal therapeutic windows. Additionally, while the present study focused on lower-limb neuromuscular parameters, future work should extend these findings to functional and systemic outcomes, such as gait performance, balance, and upper extremity coordination, to better understand the translational impact of FMV on daily activities in children with CP. Expanding investigations to include whole-body or multi-limb applications may also help clarify whether localized FMV induces broader sensorimotor adaptations across the neuromuscular system.

## Conclusion

This study investigated the effects of focal muscle vibration (FMV) at 80 Hz and 100 Hz on neuromuscular activation, fatigue, and co-contraction in children with spastic diplegic cerebral palsy (CP). The results highlight FMV’s potential as a targeted intervention with frequency-dependent effects.

Key results revealed that 100 Hz FMV elicited transient improvements in muscle activation (EMG RMS) and delayed fatigue (higher MDF) in the dominant limb, particularly in the soleus muscle, suggesting its utility for short-term spasticity management and fatigue mitigation. In contrast, 80 Hz FMV demonstrated progressive adaptations in neuromuscular coordination (CCI), indicating its potential for fostering longer-term improvements in agonist–antagonist synergy. Importantly, these between-frequency differences appear to reflect differences in the magnitude and time course of a shared, plasticity-based mechanism rather than qualitatively distinct mechanisms; given the unblinded control condition and the study’s limited statistical power, they should be regarded as hypothesis-generating and confirmed in adequately powered, sham-controlled trials. The time-dependent nature of these effects—evidenced by the return of RMS and MDF values to baseline by the 12-week follow-up—emphasizes the need for longer FMV intervention protocols to sustain therapeutic gains. These interpretations are based on EMG outcomes and require confirmation in studies measuring functional and clinical endpoints.

To our knowledge, this is the first comprehensive study to compare two FMV stimulation frequencies (80 Hz and 100 Hz) over longer (12 weeks) periods in CP children, building on prior work [61]. It offers novel insights into wearable FMV’s role in managing muscle activation, fatigue, and co-contraction. Limb-specific co-contraction differences highlight the importance of bilateral assessments in CP rehabilitation.

Limitations, including attrition, uneven group sizes, and partial follow-up data necessitate cautious interpretation. Future research should explore longer protocols (> 12 weeks), higher FMV dosages (> 10 min/muscle/visit), and functional outcomes (e.g., gait analysis). Investigating neural mechanisms via TMS/EEG and integrating FMV with task-specific training could optimize therapeutic benefits.

In conclusion, this study positions FMV as a precision tool for CP rehabilitation, providing a foundation for evidence-based, individualized strategies to enhance motor function [62].

## Data Availability

Data and materials relating to the manuscript can be made available upon reasonable request to the corresponding author.

## Abbreviations

FMV: Focal muscle vibration
CP: Cerebral palsy
sEMG: Surface electromyography
EMG: Electromyography
RMS: Root mean square
MDF: Median frequency
CCI: Co-contraction indices
GMFCS: Gross Motor Function Classification Scale
TMS: Transcranial magnetic stimulation
fMRI: Functional magnetic resonance
EEG: Electroencephalography
DF: Dorsiflexion
Sol: Soleus
TA: Tibialis anterior
LMM: Linear mixed-effects model

## References

[1] Patel DR, Neelakantan M, Pandher K, Merrick J. Cerebral palsy in children: a clinical overview. Transl Pediatr. 2020;9(Suppl 1):S125–35. 10.21037/tp.2020.01.01. 32206590 10.21037/tp.2020.01.01 PMC7082248

[2] Sadowska M, Sarecka-Hujar B, Kopyta I. Cerebral Palsy: Current Opinions on Definition, Epidemiology, Risk Factors, Classification and Treatment Options. Neuropsychiatr Dis Treat. 2020;16:1505–18. 32606703 10.2147/NDT.S235165 PMC7297454

[3] Bottcher L. Children with spastic cerebral palsy, their cognitive functioning, and social participation: a review. Child Neuropsychol. 2010;16(3):209–28. 10.1080/09297040903559630. 20209416 10.1080/09297040903559630

[4] Lowes L, Clark TS, Noritz G. Factors associated with caregiver experience in families with a child with cerebral palsy. J Pediatr Rehabil Med. 2016;9:65–72. 26966802 10.3233/PRM-160362

[5] Liu F, Shen Q, Huang M, et al. Factors associated with caregiver burden among family caregivers of children with cerebral palsy: a systematic review. BMJ Open. 2023;13:e065215. 10.1136/bmjopen-2022-065215. 37012010 10.1136/bmjopen-2022-065215 PMC10083783

[6] Pulgar S, Bains S, Gooch J, Chambers H, Noritz GH, Wright E, et al. Prevalence, patterns, and cost of care for children with cerebral palsy enrolled in medicaid managed care. J Manag Care Spec Pharm. 2019;25(7):817–22. 10.18553/jmcp.2019.25.7.817. 31232210 10.18553/jmcp.2019.25.7.817 PMC10398069

[7] Tonmukayakul U, Shih STF, Bourke-Taylor H, Imms C, Reddihough D, Cox L, et al. Systematic review of the economic impact of cerebral palsy. Res Dev Disabil. 2018;80:93–101. 10.1016/j.ridd.2018.06.012. 29981952 10.1016/j.ridd.2018.06.012

[8] Beckung E, Hagberg G. Neuroimpairments, activity limitations, and participation restrictions in children with cerebral palsy. Dev Med Child Neurol. 2002;44(5):309–16. 10.1017/S0012162201002134. 12033716 10.1017/s0012162201002134

[9] Christine C, Dolk H, Platt MJ, Colver A, Prasauskiene A, Krägeloh-Mann I. Recommendations from the SCPE collaborative group for defining and classifying cerebral palsy. Dev Med Child Neurol Suppl. 2007;109:35–8. 10.1111/j.1469-8749.2007.tb12626.x. 17370480 10.1111/j.1469-8749.2007.tb12626.x

[10] Anttila H, Autti-Rämö I, Suoranta J, et al. Effectiveness of physical therapy interventions for children with cerebral palsy: a systematic review. BMC Pediatr. 2008;8:14. 10.1186/1471-2431-8-14. 18435840 10.1186/1471-2431-8-14 PMC2390545

[11] Das SP, Ganesh GS. Evidence-based approach to physical therapy in cerebral palsy. Indian J Orthop. 2019;53(1):20–34. 10.4103/ortho.IJOrtho_241_17. 30905979 10.4103/ortho.IJOrtho_241_17 PMC6394183

[12] Steultjens EM, Dekker J, Bouter LM, van de Nes JC, Lambregts BL, van den Ende CH. Occupational therapy for children with cerebral palsy: a systematic review. Clin Rehabil. 2004;18(1):1–14. 10.1191/0269215504cr697oa. 14763715 10.1191/0269215504cr697oa

[13] Krigger KW. Cerebral palsy: an overview. Am Fam Phys. 2006;73(1):91–100. 16417071

[14] De Rinaldis M, Losito L, Gennaro L, Trabacca A. Long-term oral baclofen treatment in a child with cerebral palsy: electroencephalographic changes and clinical adverse effects. J Child Neurol. 2010;25(10):1272–4. 10.1177/0883073809357243. 20139400 10.1177/0883073809357243

[15] Kolaski K, Logan LR. A review of the complications of intrathecal baclofen in patients with cerebral palsy. In: IOS Press, NeuroRehabilitation (Vol. 22, pp. 383–395). 2007. 18162701

[16] Amin KR, et al. Remote monitoring for the management of spasticity: challenges, opportunities and proposed technological solution. IEEE Open J Eng Med Biol. 2025;6:279–86. 10.1109/OJEMB.2024.3523442. 39906269 10.1109/OJEMB.2024.3523442 PMC11793859

[17] Wilson NC, Chong J, Mackey AH, Stott NS. Reported outcomes of lower limb orthopaedic surgery in children and adolescents with cerebral palsy: a mapping review. Dev Med Child Neurol. 2014;56:808–14. 10.1111/dmcn.12431. 24673603 10.1111/dmcn.12431

[18] Steinbok P. Selective dorsal rhizotomy for spastic cerebral palsy: a review. Childs Nerv Syst. 2007;23:981–90. 10.1007/s00381-007-0379-5. 17551739 10.1007/s00381-007-0379-5

[19] Ritzmann R, Stark C, Krause A. Vibration therapy in patients with cerebral palsy: a systematic review. Neuropsychiatr Dis Treat. 2018;18(14):1607–25. 10.2147/NDT.S152543.PMID:29950843;PMCID:PMC6018484. 10.2147/NDT.S152543 PMC6018484 29950843

[20] Celletti C, Camerota F. Preliminary evidence of focal muscle vibration effects on spasticity due to cerebral palsy in a small sample of Italian children. PubMed. 2011;162(5):e125–8. 22041808

[21] Toscano M, Celletti C, Viganò A, Altarocca A, Giuliani G, Jannini TB, et al. Short-term effects of focal muscle vibration on motor recovery after acute stroke: a pilot randomized sham-controlled study. Front Neurol. 2019;19(10):115. 10.3389/fneur.2019.00115. 10.3389/fneur.2019.00115 PMC6401608 30873102

[22] Jevtic T, Zivanovic A, Loureiro RCV. Brain response to focal vibro-tactile stimulation prior to muscle contraction. In: 2016 12th international conference on intelligent environments (IE), London, UK, 2016, pp. 182–185. 10.1109/IE.2016.40.

[23] Barss TS, Collins DF, Miller D, Pujari AN. Indirect vibration of the upper limbs alters transmission along spinal but not corticospinal pathways. Front Hum Neurosci. 2021;15:617669. 10.3389/fnhum.2021.617669. 34079443 10.3389/fnhum.2021.617669 PMC8165249

[24] Viganò A, Celletti C, Giuliani G, Jannini TB, Marenco F, Maestrini I, et al. Focal muscle vibration (FMV) for post-stroke motor recovery: multisite neuroplasticity induction, timing of intervention, clinical approaches, and prospects from a narrative review. Vibration. 2023;6(3):645–58. 10.3390/vibration6030040.

[25] DeForest BA, Bohorquez J, Perez MA. Vibration attenuates spasm-like activity in humans with spinal cord injury. J Physiol. 2020;598(13):2703–17. 10.1113/JP279478. 32298483 10.1113/JP279478 PMC7517670

[26] Ashfaque M, et al. Wearable focal muscle vibration device for reducing spasticity and improving upper limb function: device co-design and results from a feasibility study in people with stroke. IEEE J Transl Eng Health Med. 2025;13:354–64. 10.1109/JTEHM.2025.3590582.

[27] Imran KN, Imran A, Irum F, Hina S, Usman R, Nitika K, Nusratnaaz S, Mads J, Kelly H, Heidi H, Simon FF, Amit NP. Wearable focal muscle vibration improves upper limb function in people with sub-acute stroke. medRxiv 2024.11.11.24317091. 10.1101/2024.11.11.24317091.

[28] Guang H, Ji L, Shi Y. Focal vibration stretches muscle fibers by producing muscle waves. IEEE Trans Neural Syst Rehabil Eng. 2018;26(4):839–46. 10.1109/TNSRE.2018.2816953. 29641388 10.1109/TNSRE.2018.2816953

[29] Marotta N, Lopresti E, Prestifilippo E, Aiello V, Mazzei M, Scozzafava L, et al. Mechanical focal vibration therapy for muscle injury recovery in a runner. Appl Sci. 2025;15(4):2022. 10.3390/app15042022.

[30] Wilkinson KA, Kloefkorn HE, Hochman S. Characterization of muscle spindle afferents in the adult mouse using an in vitro muscle-nerve preparation. PLoS ONE. 2012;7(6):e39140. 10.1371/journal.pone.0039140. 22745708 10.1371/journal.pone.0039140 PMC3380032

[31] Blum KP, Campbell KS, Horslen BC, Nardelli P, Housley SN, Cope TC, et al. Diverse and complex muscle spindle afferent firing properties emerge from multiscale muscle mechanics. Elife. 2020;28(9):e55177. 10.7554/eLife.55177. 10.7554/eLife.55177 PMC7769569 33370235

[32] Nito M, Yoshimoto T, Hashizume W, Shindo M, Naito A. Vibration decreases the responsiveness of Ia afferents and spinal motoneurons in humans. J Neurophysiol. 2021;126(4):1137–47. 10.1152/jn.00168.2021. 34495775 10.1152/jn.00168.2021

[33] Roll JP, Vedel JP, Ribot E. Alteration of proprioceptive messages induced by tendon vibration in man: a microneurographic study. Exp Brain Res. 1989;76:213–22. 10.1007/BF00253639. 2753103 10.1007/BF00253639

[34] Proske U, Gandevia SC. The proprioceptive senses: their roles in signaling body shape, body position and movement, and muscle force. Physiol Rev. 2012;92(4):1651–97. 10.1152/physrev.00048.2011. 23073629 10.1152/physrev.00048.2011

[35] Paoloni M, Giovannelli M, Mangone M, et al. Does giving segmental muscle vibration alter the response to botulinum toxin injections in the treatment of spasticity in people with multiple sclerosis? A single-blind randomized controlled trial. Clin Rehabil. 2013;27(9):803–12. 10.1177/0269215513480956. 23543344 10.1177/0269215513480956

[36] Ashfaque M, Pujari AN, Niazi IK, Amjad I, Haavik H, Farmer SF. Effectiveness of focal muscle vibrations in improving sensorimotor performance, mobility and strength in spinal cord injury population: a systematic review. BMJ Open. 2025;15(12):e110054. 10.1136/bmjopen-2025-110054. 41448695 10.1136/bmjopen-2025-110054 PMC12742131

[37] Wang H, Chandrashekhar R, Rippetoe J, Ghazi M. Focal muscle vibration for stroke rehabilitation: a review of vibration parameters and protocols. Appl Sci. 2020;10:8270. 10.3390/app10228270.

[38] Mancheva K, Rollnik JD, Wolf W, Dengler R, Kossev A. Vibration-Induced kinesthetic illusions and corticospinal excitability changes. J Mot Behav. 2016;49(3):299–305. 10.1080/00222895.2016.1204263. 27588516 10.1080/00222895.2016.1204263

[39] Lauzier L, Perron MP, Munger L, Bouchard É, Abboud J, Nougarou F, et al. Variation of corticospinal excitability during kinesthetic illusion induced by musculotendinous vibration. J Neurophysiol. 2023;130(5):1118–25. 10.1152/jn.00069.2023. 37706230 10.1152/jn.00069.2023

[40] Roll JP, Vedel JP. Kinaesthetic role of muscle afferents in man, studied by tendon vibration and microneurography. Exp Brain Res. 1982;47:177–90. 10.1007/BF00239377. 6214420 10.1007/BF00239377

[41] Reyes ML, Hernandez M, Holmgren LJ, Sanhueza E, Escobar RG. High-frequency, low-intensity vibrations increase bone mass and muscle strength in upper limbs, improving autonomy in disabled children. J Bone Miner Res. 2011. 10.1002/jbmr.402. 21491486 10.1002/jbmr.402

[42] Hoşbaş BD, Sertel M. Immediate effects of kinesio taping and vibration therapy on manual dexterity in children with unilateral spastic cerebral palsy: a randomised controlled trial. Int J Ther Rehabil. 2023;30(12):1–11. 10.12968/ijtr.2023.0047.

[43] Bloom R, Przekop A, Sanger TD. Prolonged electromyogram biofeedback improves upper extremity function in children with cerebral palsy. J Child Neurol. 2010;25(12):1480–4. 10.1177/0883073810369704. 20525944 10.1177/0883073810369704

[44] Ashfaque M, Pujari AN. Developing and characterizing a low-cost, wearable focal muscle vibration device for neurorehabilitation. In: Converging clinical and engineering research on neurorehabilitation V: proceedings of the 6th international conference on neurorehabilitation (ICNR 2024) (Vol. 1, pp. 598–602). (Biosystems and Biorobotics; Vol. 31). Springer. 2025. 10.1007/978-3-031-77588-8_117

[45] Seo HG, Oh B-M, Leigh J-H, Chun C, Park C, Kim CH. Effect of focal muscle vibration on calf muscle spasticity: a proof-of-concept study. PM&R. 2016;8(11):1083–9. 26994885 10.1016/j.pmrj.2016.03.004

[46] Rutović S, Glavić J, Cvitanović NKJ. The effects of robotic gait neurorehabilitation and focal vibration combined treatment in adult cerebral palsy. Neurol Sci. 2019;40(12):2633–4. 31203480 10.1007/s10072-019-03965-6

[47] Leonard TR, Howard JJ, Larkin-Kaiser K, Joumaa V, Logan K, Orlik B, et al. Stiffness of hip adductor myofibrils is decreased in children with spastic cerebral palsy. J Biomech. 2019;87:100–6. 30853092 10.1016/j.jbiomech.2019.02.023

[48] Darras N, Nikaina E, Tziomaki M, Gkrimas G, Papavasiliou A, Pasparakis DJ. Development of lower extremity strength in ambulatory children with bilateral spastic cerebral palsy in comparison with typically developing Controls using absolute and normalized to body weight force values. Front Neurol. 2021;12:617971. 33815249 10.3389/fneur.2021.617971 PMC8017198

[49] Marconi B, Filippi GM, Koch G, et al. Long-term effects on cortical excitability and motor recovery induced by repeated muscle vibration in chronic stroke patients. Neurorehabil Neural Repair. 2010;25(1):48–60. 10.1177/1545968310376757. 20834043 10.1177/1545968310376757

[50] Mahmood Q, Habibullah S, Babur MN. The effects of traditional massage on spasticity of children with cerebral palsy: a randomized controlled trial. J Pak Med Assoc. 2020;70:809–14. 32400732 10.5455/JPMA.24442

[51] Abd Rahman R, Baharom H. The effects of stretching, strengthening and combined interventions on lower-limb spasticity in spastic cerebral palsy: a systematic review. Int J Allied Health Sci 2026;10(1).

[52] Passos AA, Santos FO, Arida RM, Brogin JA, Faber J, López-Ortiz C, et al. Enhancing quality of life in individuals with cerebral palsy: a systematic review and meta-analysis of physiotherapy interventions. Disabil Rehabil. 2025;47(16):4040–62. 39811998 10.1080/09638288.2024.2443040

[53] Hermens HJ, Rau G, Disselhorst-Klug C. Development of recommendations for SEMG sensors and sensor placement procedures. J Electromyogr Kinesiol. 2000;10:361–74. 10.1016/S1050-6411(00)00027-4. 11018445 10.1016/s1050-6411(00)00027-4

[54] Chan ADC, Green GC. Myoelectric control development toolbox. In: 30th conference of the canadian medical & biological engineering society, Toronto, Canada, M0100, 2007.

[55] Banks CL, Huang HJ, Little VL, Patten C. Electromyography exposes heterogeneity in muscle co-contraction following stroke. Front Neurol. 2017;8:699. 10.3389/fneur.2017.00699. 29312124 10.3389/fneur.2017.00699 PMC5743661

[56] Pujari AN, Neilson RD, Cardinale M. Effects of different vibration frequencies, amplitudes and contraction levels on lower limb muscles during graded isometric contractions superimposed on whole body vibration stimulation. J Rehabil Assist Technol Eng. 2019;2019:6. 10.1177/2055668319827466. 10.1177/2055668319827466 PMC6582277 31245030

[57] Burk D, Hagbarth KE, Löfstedt L, Wallin BG. The responses of human muscle spindle endings to vibration during isometric contraction. J Physiol. 1976. 10.1113/jphysiol.1976.sp011581. 10.1113/jphysiol.1976.sp011581 PMC1309167 135841

[58] Bishop B. Vibratory stimulation—neurophysiology of motor responses evoked by vibratory stimulation. Phys Ther. 1974;54:1273–82. 10.1016/j.jelekin.2008.08.002.

[59] Lott C and Johnson MJ. Upper limb kinematics of adults with cerebral palsy on bilateral functional tasks. In: 38th annual international conference of the IEEE Engineering in Medicine and Biology Society (EMBC), Orlando, FL, USA, 2016, pp. 5676–5679. 2016. 10.1109/EMBC.2016.7592015. 10.1109/EMBC.2016.7592015 PMC11774504 28269543

[60] Silvia LP, Livia PV, Carolina SNC, Ana CC, Nelci AR. Effect of the severity of manual impairment and hand dominance on anticipatory and compensatory postural adjustments during manual reaching in children with cerebral palsy. Res Dev Disabil. 2018;83:47–56. 10.1016/j.ridd.2018.08.007. 30138846 10.1016/j.ridd.2018.08.007

[61] Boulay C, Coq JO, Sangeux M, Authier G, Ulian A, Pradines M, et al. Case report: combination of focal vibration therapy and botulinum toxin injections to treat equinus gait in a child with unilateral spastic cerebral palsy. Front Rehabil Sci. 2025;6:1454109. 10.3389/fresc.2025.1454109. 39981203 10.3389/fresc.2025.1454109 PMC11839612

[62] Stadskleiv K. Cognitive functioning in children with cerebral palsy. Dev Med Child Neurol. 2020;62:283–9. 10.1111/dmcn.14463. 32010976 10.1111/dmcn.14463

[63] Pettorossi VE, Panichi R, Botti FM, Biscarini A, Filippi GM, Schieppati M. Long-lasting effects of neck muscle vibration and contraction on self-motion perception of vestibular origin. Clin Neurophysiol. 2015;126(10):1886–900. 10.1016/j.clinph.2015.02.057. 25812729 10.1016/j.clinph.2015.02.057

[64] Fattorini L, Pettorossi VE, Marchetti E, Rodio A, Filippi GM. A review about muscle focal vibration contribution on spasticity recovery. Front Neurol. 2025;16:1579118. 10.3389/fneur.2025.1579118. 40534747 10.3389/fneur.2025.1579118 PMC12173915

[65] Filippi GM, Rodio A, Fattorini L, Faralli M, Ricci G, Pettorossi VE. Plastic changes induced by muscle focal vibration: a possible mechanism for long-term motor improvements. Front Neurosci. 2023;17:1112232. 10.3389/fnins.2023.1112232. 36908788 10.3389/fnins.2023.1112232 PMC9992721

[66] Goodwin GM, Mccloskey DI, Matthews PBC. The contribution of muscle afferents to keslesthesia shown by vibration induced illusionsof movement and by the effects of paralysing joint afferents. Brain. 1972;95(4):705–48. 10.1093/brain/95.4.705. 4265060 10.1093/brain/95.4.705

[67] Rosenkranz K, Rothwell JC. The effect of sensory input and attention on the sensorimotor organization of the hand area of the human motor cortex. J Physiol. 2004;561:307–20. 10.1113/jphysiol.2004.069328. 15388776 10.1113/jphysiol.2004.069328 PMC1665339

